# Improving Learners' Comfort With Cesarean Sections Through the Use of High-Fidelity, Low-Cost Simulation

**DOI:** 10.15766/mep_2374-8265.10878

**Published:** 2020-02-14

**Authors:** Tatiana Acosta, Jill Marie Sutton, Sarah Dotters-Katz

**Affiliations:** 1 Resident, Department of Obstetrics and Gynecology, Duke University Medical Center; 2 Clinical Associate Professor, Department of Obstetrics and Gynecology, Brody School of Medicine at East Carolina University; 3 Director of Undergraduate Medical Education, Department of Obstetrics and Gynecology, Brody School of Medicine at East Carolina University; 4 Assistant Professor, Department of Obstetrics and Gynecology, Duke University School of Medicine; 5 Director of Undergraduate Medical Education, Department of Obstetrics and Gynecology, Duke University School of Medicine

**Keywords:** Simulation, Model, Cesarean Section, C-Section, Cesarean Delivery, Obstetrics, OB/GYN

## Abstract

**Introduction:**

Several studies have demonstrated effective simulation-based training for laparoscopic procedures in OB/GYN, but limited simulation curricula exist for abdominal procedures, particularly cesarean sections (CSs).

**Methods:**

We developed a high-fidelity modification of an existing CS model costing about $25 and incorporated it into a 90-minute teaching simulation event for medical students and OB/GYN residents in a single academic program. The simulation included a structured curriculum, pre-/postsimulation surveys, a surgical instrument review, a mannequin with the CS model containing a fetus in breech position, and live video streaming. Our surveys assessed participants' comfort with the procedure and its related components on a 5-point scale, and we used a paired *t* test to analyze our data.

**Results:**

Twenty-two learners (eight third-year medical students, one fourth-year medical student, three first-year residents, four second-year residents, one third-year resident, four fourth-year residents, and one unknown level) participated in this simulation. We found a statistically significant improvement in perceived CS instrument knowledge, suturing skills, and satisfaction with the model among all participants. Only third-year medical students had a statistically significant increase in comfort level in performing a CS after the simulation. Video streaming engaged a wider audience, but poor lighting and audio limited its efficacy.

**Discussion:**

Using this simulation model at the end of medical school or early in residency may have the greatest positive effect on resident comfort with CSs. This low-cost and versatile model can be used across educational settings, including OB/GYN interest group activities, intern boot camp, and interprofessional emergency drills.

## Educational Objectives

By the end of this activity, learners will be able to:

1. Name and identify the most common surgical instruments for performing a cesarean section (CS).
2. Label each anatomical layer encountered during a CS with the correct instrumentation for each layer.
3. Demonstrate the correct suturing technique to close each anatomical layer.
4. Exhibit increased comfort in performing a CS.

## Introduction

Cesarean sections (CSs) are one of the most common surgical procedures in obstetrics, accounting for about one in three deliveries.^1^ Resident learners begin performing CSs in their first or second year of training and commonly perform a CS for the first time on a real patient without any prior hands-on practice. In contrast, many graduate and undergraduate training programs have incorporated vaginal delivery simulations with high-fidelity pelvic models or even full mannequins such as the Noelle to increase learners' comfort level with vaginal deliveries.^2–4^

Many OB/GYN residency programs have adopted simulation training in laparoscopic procedures,^5–7^ but limited simulation training curricula exist in open abdominal procedures, particularly CSs. Vellanki and Gillellamudi^8^ compared comfort level between learners who participated in a simulation under guided instruction using a low-cost CS model versus learners with no simulation-based learning and found that participants in the simulation were more comfortable assisting with a cesarean delivery, but those authors did not include a step-by-step guide to replicate their simulation. Sampson, Renz, and Wagner^9^ developed a low-cost model for simulation of a perimortem cesarean delivery and included instructions on how to build their model. However, the simulation did not mention providing the opportunity to practice closing every abdominal wall layer with the appropriate suture, which is part of fully completing a CS. More expensive, high-fidelity CS models have also been used. Yu, Wilson, and Janssens^10^ developed a curriculum using a high-fidelity model called Desperate Debbie to simulate the cesarean delivery of a deeply impacted fetal head, but their curriculum did not focus on development of basic surgical skills and comfort in performing CSs. Similarly, Rajakumar et al.^11^ used a high-fidelity model called SimMom to simulate an emergent cesarean delivery for an umbilical cord prolapse, but the focus was on communication and team dynamics rather than on the surgical procedure itself.

Stickrath and Alston^12^ developed a novel low-fidelity abdominal hysterectomy model available in *MedEdPORTAL* to improve residents' confidence with the procedure, as it has become a less commonly performed surgical option, but no curricula are available on CS simulation with a low-cost, high-fidelity model. The goal of this project was to develop a multimedia-enhanced, resident-to-learner teaching CS simulation event where learners participated within a high-reality framework to become more comfortable performing a CS. By including learners from multiple levels, we also sought to give upper-level teaching residents the opportunity to practice teaching a surgical skill in a low-stress setting. This simulation is not limited to training in OB/GYN. Learners from family medicine programs could use this curriculum to prepare as surgical assistants or primary surgeons. The simulation is also applicable to interprofessional education with emergency medicine residents, anesthesia residents, certified nurse anesthetist students, and nursing students.

## Methods

### Development

We developed this simulation as a quality improvement project in education. The Institutional Review Board at East Carolina University granted it exemption (UMCIRB 18-001032). The simulation (outlined in Appendix A) was designed to last about 90 minutes, with time for a presimulation survey, a 10-minute presentation discussing the patient scenario and reviewing the surgical instruments, the simulation itself, and a postsimulation survey. The cohort to complete the curriculum included third-year medical students, a fourth-year OB/GYN acting intern, and all residents present during a protected Wednesday afternoon didactics session at the East Carolina University OB/GYN Residency Program at Vidant Medical Center. The OB/GYN clerkship director and the first author led the simulation.

### Equipment/Environment

We developed a high-fidelity modification of an existing low-cost CS model by Dr. Meg O'Reilly and her team at Oregon Health & Science University.^13^ Our modifications to the model included using a different plastic box that contained a handle that acted as the pubic symphysis and creating a uterine cavity to contain an amniotic sac, fetus, and placenta. All of the supplies could be purchased at commonly found craft stores, superstores, or online. The unit cost per model was about $25, and each model could be used twice. The upfront cost, however, was closer to $100. Appendix B lists the necessary supplies, approximate cost of each, and step-by-step instructions and images of how to build the model. Preparing all materials and cutting the various fabrics took about 2–3 hours. However, model assembly was much faster and took about 10–15 minutes.

We also developed a complementary curriculum to go along with the model. This included an instructional PowerPoint describing common CS surgical instruments (Appendix C), an instructional Word document outlining the steps and instruments called during a CS along with the steps of a surgical time-out (Appendix D), and written simulation instructions containing a clinical vignette describing a fetus in breech position (Appendix A). We also created pre- and postsimulation surveys (Appendices E and F, respectively) to assess the participants' experience, as described in the Assessment section, below.

One week prior to the simulation, learners received an instructional email containing the surgical instruments PowerPoint (Appendix C) and CS steps and time-out (Appendix D). The email instructed them to review the documents prior to coming to the simulation.

The basic equipment included the CS model, a rectangular table for the model, an operating room instrument table, a standard CS tray, and standard suture material. Appendix B lists the necessary and optional equipment. Addition of optional equipment would make the simulation more realistic. A few days prior to the simulation, we reserved the simulation equipment from the School of Medicine's simulation center. Many of the supplies, such as the surgical drape, IV pole and fluid, electrosurgical pencil, and suction tip and tubing, could be used more than once. The OB/GYN clerkship director and a surgical scrub technician facilitated obtaining the CS tray.

An hour prior to the scheduled simulation time, we arranged a lecture classroom to mimic an operating room environment. We placed the CS model in the middle of the rectangular table, and the torso and head of a mannequin were placed superior to the model to complete our patient. Two chairs were placed at the end of the table by the patient's head to allow anesthesia and supporting personnel to sit. An IV pole with a bag of fluid was placed on the patient's left side. Our surgical scrub technician set up the instrument and Mayo tables by the patient's feet. Third-year medical students in their OB/GYN rotation created the video system. First, they created tape slings to hang an iPad from the existing ceiling projector. Next, the table was arranged so that the iPad's camera was focused directly on the patient's abdomen—that is, the CS model. Any tablet with video and live-chat capabilities could be used. Finally, the students initiated a video chat between the iPad and the computer connected to the projector so that the CS model was projected on a large white screen. Subsequently, the students prepped and draped the mannequin in the normal fashion using surgical drapes (Appendix G contains an image of the final product). The OB/GYN clerkship director used this as an opportunity to teach the medical students sterile technique. However, this step could also be performed at the start of the CS procedure (see the following).

### Personnel

Two preceptors, one faculty member and the primary author, who at the time was a medical student, were present to facilitate the simulation. However, the ratio of preceptors to the team of learners directly using the CS model in the simulation should be 1:1 or 1:2, and the preceptor should be a faculty member or upper-level resident. Participant roles were defined prior to starting the simulation, and one of the two preceptors handed out index cards to the appropriate individuals with their assigned roles. Eight participants, including OB/GYN residents and medical students, volunteered to be directly involved in the simulation and were assigned the following roles: one first-year resident as the primary surgeon, one fourth-year resident as the primary assistant, one third-year resident as the attending, one third-year medical student as the medical student, one fourth-year resident as the surgical scrub technician, one second-year resident as the anesthesiologist/certified nurse anesthetist, and two third-year medical students as the baby nurse and support person. Appendix A describes each role in detail.

• *Essential roles:* primary surgeon, primary or first assistant, attending or teaching surgeon.
• *Nonessential roles:* medical student, surgical scrub technician, anesthesiologist/certified nurse anesthetist, baby nurse, support person.

### Implementation

Prior to entering the simulation room, we asked learners to complete the presimulation survey. At this time, the OB/GYN clerkship director handed out index cards to the eight participants with their assigned roles. Participants also picked up a copy of the CS steps and time-out document (Appendix D) prior to entering the room. Once everyone had entered the room, we began the simulation by presenting the clinical vignette. The CS team was then asked to step out to perform a simulated surgical scrub, and everyone else remained in the room. Upon the CS team's return, the surgical scrub technician gowned first in a sterile manner, then helped the other team members with the gown and gloves as they entered the room, exactly as would happen during a case. We then began reviewing the CS instruments one by one from the instrument table. Each instrument was projected on the large screen by bringing it under the iPad, which was serving as the projector, and the audience was asked to call out the instrument's name.

The CS team gathered around the patient, who had already been prepped and draped in the normal fashion, as mentioned above, although the prepping and draping could have been done at this time instead. The suction and electrosurgery pencil were set up as usual, and then the primary surgeon performed the Allis clamp test. The time-out was performed. The members of the surgical team performed their roles with permission to ad lib according to their experiences. The first assistant and attending assisted the primary surgeon in performing the procedure from skin incision to delivery of the neonate to skin closure. The critical actions checklist (Appendix H) lists the essential steps to be performed during the simulation. The procedure itself took about 1 hour to complete. This could be longer or shorter depending on the learner level and surgical skills. Any team members not directly involved in the procedure sat in chairs around the classroom and watched the simulation projected on a white screen from the multimedia setup.

### Assessment

We developed pre- and postsimulation surveys by drawing on the expertise of the OB/GYN clerkship director and student surveys she had previously used. The presimulation survey (Appendix E) consisted of five questions that participants answered using a 5-point scale. Four questions asked the learners to rate their baseline comfort level with knowing the surgical instruments, naming the anatomical layers encountered in a CS, performing the procedure, and using appropriate suturing technique in closing each anatomical layer. The last question inquired about the participants' expectation of the model. Upon completion of the simulation, the learners were asked to complete the postsimulation survey (Appendix F). The postsurvey repeated the comfort-based questions from the presurvey, and participants additionally were asked to rate the model and provide feedback. We thus measured our objectives by assessing the learners' perceived comfort with each topic addressed by the survey questions, which were based on our stated objectives. The surveys remained anonymous, and learners were only asked to write down their level of training.

We used a paired *t* test and Stata software (StataCorp, College Station, Texas) to calculate the mean score for each survey question by participant education level. We used a 95% confidence interval with a *p* value of .05 to define statistical significance. We also qualitatively analyzed the participants' subjective comments.

### Debriefing

Once the simulation ended (i.e., the skin was closed), learners had a chance to debrief with the team. The OB/GYN attending leading the simulation facilitated the discussion, which included questions such as “What do you think you did well?”, “What did you find challenging?”, “What did you find particularly helpful?”, and “What do you need to improve on?” Additional questions addressing key concepts of a CS were also discussed (Appendix I). The instructor then reviewed the critical actions checklist with each learner, acknowledged the learner's strengths, and identified areas for improvement. Learners were encouraged to review the handout containing the steps of a CS prior to performing the procedure on a real patient. Learners were then asked to complete the postsimulation survey.

## Results

A total of 22 learners (eight third-year medical students, one fourth-year medical student, three first-year residents, four second-year residents, one third-year resident, four fourth-year residents, and one unknown training level) participated in the simulation. We conducted the simulation at the beginning of the academic year, so each learner was in a new level of training. Data from the presimulation survey were missing for four participants. Three of these four participants were a third-year medical student, a fourth-year medical student, and a second-year resident. These three individuals could not be matched to their corresponding postsimulation surveys because no identifiable information was used, but they were not discarded because this would have led to incorrectly discarding data for a participant who had matching pre- and postsimulation surveys. Since one participant did not write down the year of training, the participant's data could not be used in the analysis and were discarded. Therefore, survey responses from 21 learners were used in the data analysis. The mean baseline comfort level for all learners with regard to instrument knowledge, knowledge of anatomical layers, comfort performing a CS, and comfort with suturing skills was fair to very good (Table 1). We found a statistically significant improvement in perceived CS instrument knowledge, suturing skills, and satisfaction with the model among all participants.

**Table 1.** Mean Difference in Scores Among All Participants (*N* = 21)

Topic	Mean Baseline Score^a^	Mean Postsimulation Score^a^	Mean Difference (95% Confidence Interval)	*p*
Instrument knowledge	2.8	3.5	0.7 (0.2 to 1.3)	.01
Knowledge of anatomical layers	3.3	3.8	0.5 (−0.2 to 1.2)	.07
Comfort performing a cesarean section	2.4	2.8	0.3 (−0.2 to 1.1)	.1
Comfort with suturing skills	2.7	3.6	0.9 (0.1 to 1.7)	.02
Model rating (expectations)	4.2	4.7	0.6 (0.1 to 1.0)	.01

a Rated on a 5-point scale (1 = *poor*, 5 = *excellent*).

Subanalysis of third-year medical student surveys revealed a statistically significant improvement in instrument knowledge, comfort performing a CS, comfort with suturing skills, and model rating (Table 2). Table 3 demonstrates the difference in mean baseline comfort level in performing a CS across education levels and the degree of change in comfort level after the procedure. Since only one fourth-year medical student and one third-year resident participated in the simulation, we did not include their data in Table 3. Those in their earlier years of training (third-year medical students and first-year residents) had the lowest comfort level but also had the largest absolute change in comfort performing a CS after the simulation.

**Table 2.** Mean Difference in Scores Among Third-Year Medical Students (*N* = 8)

Topic	Mean Baseline Score^a^	Mean Postsimulation Score^a^	Mean Difference (95% Confidence Interval)	*p*
Instrument knowledge	1.6	3.1	1.6 (0.8 to 2.3)	<.001
Knowledge of anatomical layers	2.6	3.7	1.1 (−0.4 to 2.7)	.06
Comfort performing a cesarean section	1.1	2.6	1.4 (0.9 to 1.9)	.001
Comfort with suturing skills	1.6	3.6	2.0 (0.9 to 3.1)	.002
Model rating (expectations)	4.1	4.9	0.7 (0.0 to 1.4)	.02

a Rated on a 5-point scale (1 = *poor*, 5 = *excellent*).

**Table 3.** Mean Comfort Level in Performing a Cesarean Section by Education Level (*N* = 19)

Education Level	Presimulation Comfort Level^a^	Postsimulation Comfort Level^a^	Mean Difference (95% Confidence Interval)	*p*
Third-year medical student (*n* = 8)	1.1	2.5	1.4 (0.9 to 1.9)	.0004
First-year resident (*n* = 3)	1.3	2.7	1.3 (−0.1 to 2.8)	.06
Second-year resident (*n* = 4)	3.3	3.3	−0.3 (−1.8 to 1.1)	.42
Fourth-year resident (*n* = 4)	4.3	4.3	0.0 (−1.3 to 1.3)	1.00

a Rated on a 5-point scale (1 = *poor*, 5 = *excellent*).

As noted in Tables 1 and 2, all participants had a very good expectation of the CS model, and their final rating statistically significantly surpassed their expectation. Table 4 summarizes the comments by theme and the prevalence of each theme. Notably, the majority of comments (41%) were positive and included “I loved this simulation” and “Very helpful!” Constructive feedback focused on more opportunities to participate, better audio, and more teaching. Not shown, only one comment wished for a shorter simulation.

**Table 4.** Participant Feedback (*N* = 21)

Theme	Percentage of Total Comments	Comments
Usefulness/positive reaction	41	•“Very helpful!” •“I loved this simulation.”
More participation	13	•“Would have been nice to participate” (first-year resident). •“More crowd involvement” (fourth-year resident).
More teaching	14	•“Explanation of suture choices for each layer.” •“Get residents/attendings to teach more.”
Better audiovisual	18	•“Could not always hear what they were doing.” •“Microphones and holding instruments up to camera when reviewing prior to simulation.”
Greater fidelity	14	•“More body fluids!” •“Add blood.”

## Discussion

By offering a step-by-step guide for assembling a CS model and running the simulation, this curriculum adds to the body of literature on incorporating high-fidelity, low-cost cesarean delivery simulation into learners' training. This CS simulation curriculum demonstrated a positive trend in comfort level among third-year medical students and interns performing a CS, but only third-year medical students had a statistically significant improvement in their comfort level. We found a statistically significant positive change in comfort with knowledge of CS instruments and suturing skills among all participants, specifically among third-year medical students. It is possible that the third-year medical students biased the results obtained for all learners against the null. Due to the small sample size, we could not perform subset analysis for the other levels of training.

Participants were very receptive to the simulation. Their experience surpassed their expectation, which highlights learners' acceptance of simulation for procedural training and is consistent with other studies.^14,15^ Using this simulation model early in residency training is likely to have the greatest positive effect on increasing the comfort level of trainees performing CSs. Upper-level medical students interested in OB/GYN and/or first-year residents are more likely to benefit from this simulation than are upper-level residents who have already gained experience in CSs. However, upper-level residents can use the simulation as an opportunity to refine their teaching skills.

This study has several strengths. The real-life simulation allowed for a low-stress teaching environment. Willis et al.^16^ found that in laparoscopic simulation skill training, proficiency-based training, which allows for multiple attempts with immediate feedback, resulted in better trainee performance compared to traditional time- or repetition-based training. Currently, the norm is for residents to gain experience and competency in performing CSs through repetition on real patients. However, just as with laparoscopic simulation, CS simulation can provide the opportunity for repeated attempts and real-time feedback in a low-stakes environment.

Next, we used a comprehensive curriculum that included an instrument review PowerPoint and handout containing the steps of a CS and time-out, which allowed learners to become acquainted with the procedure and appropriate instruments prior to the simulation. Rather than practicing suturing skills in silos, learners used the appropriate suture material and suturing technique to close each anatomical layer. Another strength is that this high-fidelity model is inexpensive to make and can be used twice by flipping the uterus over to use the uncut side and turning the abdominal wall upside down to also use the uncut side. Expensive CS models such as C-Celia^17^ cost thousands of dollars and may not be a feasible purchase for most residency programs. Additionally, because our clinical vignette has a fetus in breech position, the participants also learn the standard maneuvers for breech extraction. Finally, the knowledge participants acquire should be transferable to the operating room. Although knowledge recall has been shown to be greatest when the environment mimics the setting in which the knowledge was acquired and although our simulation environment was highly realistic, Coveney, Switzer, Corrigan, and Redmond^18^ found no difference in knowledge recall among medical students who acquired knowledge in the classroom versus the operating room.

Since this first simulation, we have made a few changes based on feedback. Amniotic fluid was added to the model to make it more realistic. We also added more time to answer questions prior to starting the procedure. In addition, simulation sessions have included smaller teams of two to four participants and multiple simulations running at the same time, requiring the use of multiple CS trays. Two preceptors were needed when four simulations were running at the same time, where upper-level residents served as the preceptors. Finally, we developed an objective assessment of the participants' knowledge before and after the simulation using the evidence-based recommendations for performing CSs by Dahlke et al.^19^ and consulting with subject matter experts. This objective assessment consists of a pre- and postsimulation quiz with multiple-choice, matching, and free-response questions that ask learners to name different surgical instruments, list in order the abdominal wall layers encountered during a CS, match the surgical instrument with its corresponding abdominal wall layer, select the image that represents the most commonly used hysterotomy, and name the type of suturing technique used for each of the listed layers of the abdomen. So far, participants have objectively shown improvement in their knowledge, but our sample size remains small for any formal data analysis.

One application of this model is in residency preparatory curricula for graduating medical students entering OB/GYN. Future studies need to examine the effectiveness of this model in skill retention from the time the trainee performs the CS simulation as a fourth-year medical student to the time the trainee performs his or her first CS on a real patient. This model could be used during orientation of first-year residents, as well as for milestone assessment, where the critical actions checklist (Appendix H) could be part of an objective structured clinical examination for late-year interns or early second-year residents. In addition, attending physicians could be asked to formally evaluate learners' skill and confidence after their first actual CS to provide an additional objective measure. This CS model could also easily be used for interprofessional simulation with nursing students, surgical scrub students, and nurse anesthetist students. Beyond the development of technical skills, this model, along with an appropriately adapted curriculum, has the potential to help with nontechnical skills such as communication and teamwork, decision making, and leadership^20^ when used in interprofessional simulation settings such as emergency drills and perimortem CS simulations.

Limitations of this study included a small sample size. As a result, we could not perform subset analysis for first-year residents, who are among the most likely to benefit from the simulation. However, the absolute magnitude in the change in comfort level was comparable to that experienced by our third-year medical students. Grouping all levels of training in our data analysis of each survey topic likely skewed the results toward the null, as we did not find a statistically significant change in comfort level in performing CSs among all participants, but subset analysis revealed a statistically significant change among third-year medical students. We reasonably expect that upper-level residents are comfortable performing a CS and do not gain additional comfort by performing a simulation on a model. We also had three missing data values from the presimulation survey, which also likely skewed our results given the small sample size. Furthermore, we lack data to conclude that an increase in comfort will translate into improved competence. Another limitation is that to conserve anonymity, we compared pre- and postsurvey means for each level of training as an aggregate of participants without specifically pairing a particular postsurvey result with its presurvey equivalent. In the future, we could assign codes to the surveys to assess participants individually while retaining anonymity.

Although we included live video streaming to engage a wider audience than those directly participating in the simulation, subjective feedback revealed that this was not as useful as expected due to poor audio and lighting. However, live video streaming is not a necessary component of the simulation as long as more learners have the opportunity to be directly involved with the simulated surgical procedure. In the future, video may be used to record learners' performance to provide individualized direct feedback. We have run the simulation without this aspect in smaller groups and have noticed no difference in learner satisfaction.

Time was also a limitation. We carried out the simulation only once for the results listed earlier. Although learners on the periphery seemed to also benefit from the simulation by watching on the projected screen, not all first-year residents had the opportunity to act as primary surgeons, so our data do not fully capture the degree of effectiveness of our simulation curriculum and model. Finally, our simulation included measurement of only subjective rather than objective data. However, our goal was to increase participants' comfort with the procedure rather than to measure their skill level. However, an area of future study consists of finding an objective measure of the effectiveness of this curriculum.

In summary, this CS simulation model and curriculum were effective in increasing comfort level in performing the procedure among lower-level participants. We have found this simulation to be useful in myriad settings. It has the potential to help learners not only acquire technical skills but also acquire nontechnical skills, which are essential in providing quality care to our patients.

## Appendices

A. Simulation Case.docx
B. CS Model Assembly and Materials.docx
C. Surgical Instruments.pptx
D. CS Steps and Time-out.docx
E. Presimulation Survey.docx
F. Postsimulation Survey.docx
G. Simulation Images.docx
H. Critical Actions Checklist.docx
I. Debriefing Materials.docx All appendices are peer reviewed as integral parts of the Original Publication.
