# Supplementary material for: Improving Learners' Comfort With Cesarean Sections Through the Use of High-Fidelity, Low-Cost Simulation
Source: MedEdPORTAL. 2020 Feb 14;16:10878. doi: 10.15766/mep_2374-8265.10878 (PMC7062555; doi:10.15766/mep_2374-8265.10878)
Supplement: Supplementary file 1 — A. Simulation Case.docx B. CS Model Assembly and Materials.docx C. Surgical Instruments.pptx D. CS Steps and Time-out.docx E. Presimulation Survey.docx F. Postsimulation Survey.docx G. Simulation Images.docx H. Critical Actions Checklist.docx I. Debriefing Materials.docx [file mep-16-10878-s001.zip › A. Simulation Case.docx]

| **Appendix A: Cesarean Section Simulation Case**  **SIMULATION CASE TITLE:** Improving Cesarean Section Skills Through the Use of High-Fidelity Simulation  **AUTHORS:** Tatiana Acosta, MD, MPH; Jill Marie Sutton, MD, FACOG; Sarah Dotters-Katz, MD, MMHPE | |
| --- | --- |
| **PATIENT NAME: Amy Nitis**  **PATIENT AGE: 31 year-old female**  **CHIEF COMPLAINT: Leakage of fluid** | |
|  | |
| **Brief narrative description of case** | 31-year-old G1P0 at 37 weeks and three days by her last menstrual period consistent with a 12-week ultrasound. She spontaneously ruptured her membranes last night. She has since developed chorioamnionitis and her fetus is in breech position. Your goal is to perform every step of a primary Cesarean section on this patient from skin incision to skin closure, delivering the infant using standard breech maneuvers. |
| **Primary Learning Objectives** | By the end of this activity, the learner will:   1. Name and identify the most common surgical instruments for performing a Cesarean section 2. Label each anatomical layer encountered during a Cesarean section with the correct instrumentation for each layer 3. Demonstrate the correct suturing technique to close each anatomical layer 4. Exhibit increased comfort in performing a Cesarean section |
| **Critical Actions** | Preparation   - Apply mask, shoe coverings and hair covering - Perform a surgical scrub - Get gowned and gloved in sterile fashion - Prep and drape the patient in standard fashion (guided by instructor)   -Learner will go through the motions of cleaning the abdomen per the institution’s protocol  -Learner will drape the patient using the appropriate surgical drape  Procedure: Abdominal wall entry and delivery   - Perform a surgical time-out (Appendix D) - Perform the Allis test - Call for a knife with #10 blade, verbalize “incision” to mark the start of the procedure and perform a Pfannenstiel skin incision - Call for two medium Richardsons, a pair of Ferris-Smiths, and curved Mayos. Dissect the fascia - Call for two Kochers and elevate the fascia off the rectus abdominis muscle - Call for curved Mayos and dissect the remaining fascia off the rectus abdominis muscle - Call for two Kelly clamps and Metzenbaum scissors to grasp and dissect the peritoneum. Extend the dissection bluntly - Call for a large Richardson and a bladder blade. Retract back the abdominal wall and protect the bladder - Call for Russian forceps and Metzenbaum scissors. Create a bladder flap (optional) - Call for a knife with #10 blade and make a low transverse hysterotomy incision - Call for an Allis and rupture the amniotic sac - Remove the bladder blade and deliver the fetus using standard breech maneuvers - Call for plastic clamps and scissors and cut the cord - Remove the placenta via manual expression of the uterus and gentle traction on the umbilical cord - Call for a Pennington to remove any trailing membranes from the placenta (may go through the motions for this step as no trailing membrane exists in the simulation model) - Call for a sponge or dry lap to clear out the uterus of all debris   Procedure: Hysterotomy and abdominal wall closure   - Call for a pair of Russians and 0-Monocryl and close the hysterotomy using a running locking suture technique - Call for two hemostats to tag the suture tails (optional) or call for suture scissors to cut the suture - Inspect for hemostasis - Call for two Kocher clamps and elevate the fascia to inspect for hemostasis - Call for a medium and a small Richardson retractor, a pair of Ferris-Smiths, and 0 or 1.0 PDS. Close the fascia using a simple running suture technique. Call for suture scissors and cut the suture - Obtain the Bovie. Inspect for hemostasis of the subcutaneous tissue and use electrosurgery where needed to establish hemostasis (may go through the motions for this step as no bleeding will be present) - Call for Adsons and 3.0 Vicryl or Monocryl to close the subcutaneous space (when thickness is 2 cm or greater). Call for suture scissors to cut the suture - Call for Adsons and 4.0 Vicryl to close the skin in a subcuticular fashion |
| **Learner Preparation** | A week prior to the case simulation, the learner will receive an e-mail containing a PowerPoint of common surgical instruments found in a Cesarean section tray (Appendix C), a handout containing the surgical timeout and steps of a Cesarean section (Appendix D), and an optional link to Dr. O’Reilly’s C-section model in action for students to review the steps of a C-section (https://vimeo.com/83562510). |

| Initial Presentation | |
| --- | --- |
| **Initial vital signs** | Temperature 39°C, blood pressure 135/80, heart rate 110, respiratory rate 12, SpO2 100%. |
| **Overall Appearance** | When the learner enters the room, the patient is on the surgical table with regional anesthesia. She looks nervous. |

| **Actors and roles in the room at case start** | The instructor leading the simulation should be someone who can confidently perform a cesarean delivery independently. In our experience, this is usually an OB/GYN attending, a fellow, or a chief resident.  Essential roles   - Primary Surgeon: An upper-level medical student or intern will best fill this role. - Primary or First Assistant: An upper-level medical student, intern, or upper-level resident can fill this role. This is the second most valuable role for the learner and should be prioritized over other roles if learner number is limited. - Attending or Teaching Surgeon: A upper-level resident will best fill this role if running the simulation with a graduate trainee as primary surgeon. An end-of-year intern can also fill this role *if working with medical students.*   Non-essential roles   - Medical student: A higher-level learner or junior student can fill this role. This participant performs the roles of a typical medical student at your institution. - Surgical scrub technician: An upper level medical student, intern, or resident can best fill this role. This participant stands next to instrument table to hand instruments as the primary surgeon or assistant calls for them. - Circulating nurse: Anyone with surgical experience can fill this role. This participant helps with timeout, does not stand at surgical table, and can give good feedback about communication and teaching. - Baby nurse: Anyone can fill this role. This participant catches the baby after the cord is cut. - Anesthesia/ certified nurse anesthetist: Anyone with surgical experience can fill this role. This participant stands above the drape at head of the patient and has a good view of the simulated procedure. - Support person: Anyone can fill this role. This participant sits behind the drape and asks questions that family member might ask to make this more “realistic”. | | |
| --- | --- | --- | --- |
| **HPI** | The following information is shared with the learners. Since the focus is on the surgical procedure, the learner does not need to gather additional information.  Amy Nitis, is a 31-year-old G1P0 at 37 weeks and three days by her last menstrual period consistent with a 12-week ultrasound with a pregnancy complicated by chronic hypertension who presented to triage on a Friday morning complaining of leakage of fluid since the previous night. She reports occasional contractions but nothing consistent. She is not feeling well. She has missed her last two OB appointments. On physical exam, she is febrile, has uterine tenderness, and the fetus is tachcyardic. Her speculum exam is consistent with ruptured membranes by pooling, positive Nitrazine, and ferning. She also meets criteria for chorioamnionitis. You decided that the next best step is delivery. Her cervical exam reveals 2 cm dilation, 30% effacement, and fetal station at -3. You perform a bedside ultrasound and confirm that the fetus is breech. You decide to proceed with a primary Cesarean section for fetal malpresentation in the setting of chorioamnionitis. | | |
| **Past Medical/Surgical History** | **Medications** | **Allergies** | **Family History** |
| None | None | None | Mother with diabetes |
| **Physical Examination** | | | |
| **General** | Appears anxious, but in no acute distress | | |
| **HEENT** | Moist mucosal membranes | | |
| **Neck** | No thyromegaly noted | | |
| **Lungs** | Clear to auscultation bilaterally | | |
| **Cardiovascular** | Regular rate and rhythm; no murmurs | | |
| **Abdomen** | Gravid, tender to palpation over fundus | | |
| **Neurological** | Grossly intact | | |
| **Skin** | No lesions | | |
| **GU** | Normal external genitalia  Speculum exam: positive pooling, nitrazine, and ferning  Cervical exam: 2 cm dilation, 30% effacement, and fetal station at -3 | | |
| **Psychiatric** | Normal mood and affect | | |

| Instructor Notes - Changes and CASE Branch Points | | |
| --- | --- | --- |
| **Time point** | **Change in Case** | **Additional Information** |
| Delivery of fetus | Hysterotomy is not wide enough and participant encounters difficulty delivering the fetus | Instructor guides participant in how to extend a uterine incision |
| Hysterotomy incision closure | Hysterotomy is not hemostatic | Primary surgeon is instructed to perform one figure-of-eight suture |

**Ideal Scenario Flow**

Before the learners enter the simulation room, they fill out a pre-simulation survey and pick up a copy of the CS steps and time-out document (Appendix D). At this time, the instructor passes out index cards to the participants explaining their role in the simulation: primary surgeon, first assistant, attending, medical student, surgical scrub technician, circulating nurse, baby nurse, certified nurse anesthetist, and support person. The instructor then introduces the simulation to learners by reading the patient scenario. Next, learners review the instrument names. One by one, the instructor picks up an instrument and places it under the tablet device so that learners sitting in the periphery can view the instruments on the projector screen. After the instrument review, the learners who will be will be performing the surgical simulation step out to put on the appropriate attire and perform a surgical scrub. The learners enter the room and approach the patient. They place the surgical drape in standard fashion and perform the time-out. They then start performing the CS, calling for the appropriate instrument by name and following each step of the procedure in the correct order as noted in the Critical Actions Checklist (Appendix H). The first assist will guide the primary surgeon in performing the CS. The instructor will oversee the simulation and supplement the resident-to-learner teaching.

**Anticipated Management Mistakes**

1. Difficulty delivering the fetus: Since the material for the uterus is tougher than a real uterus, learners may encounter difficulty delivering the fetus. This, however, provides an opportunity for the instructor or first assist to teach standard breech maneuvers and how to approach difficult deliveries including deciding when to extend the hysterotomy.
2. Incorrect instrument call: Since this simulation is performed with lower-level learners, we expect that they will encounter come trouble recalling instrument names. For this reason, we included an instrument review prior to the start of the simulation. Learners receive real-time feedback as they perform the simulation.
3. Difficulty suturing: As with any skill, suturing takes practice and it is a slow process in the beginning. Learners must have sufficient time to complete the simulation in order to perform every step and receive the appropriate feedback.
