# Supplementary material for: Improving Learners' Comfort With Cesarean Sections Through the Use of High-Fidelity, Low-Cost Simulation
Source: MedEdPORTAL. 2020 Feb 14;16:10878. doi: 10.15766/mep_2374-8265.10878 (PMC7062555; doi:10.15766/mep_2374-8265.10878)
Supplement: Supplementary file 1 — A. Simulation Case.docx B. CS Model Assembly and Materials.docx C. Surgical Instruments.pptx D. CS Steps and Time-out.docx E. Presimulation Survey.docx F. Postsimulation Survey.docx G. Simulation Images.docx H. Critical Actions Checklist.docx I. Debriefing Materials.docx [file mep-16-10878-s001.zip › B. CS Model Assembly and Materials.docx]

**Appendix B:** Cesarean Section Model Assembly and Simulation Materials

| **Materials**  **(anatomical layer or structure)** | | **Cost (Unit Cost Per Model)** | **Assembly** | **Images**  Images are author owned |
| --- | --- | --- | --- | --- |
| Layers of Abdominal Wall | Faux suede or thin leather fabric (skin) | $8.99/yard ($0.99) | Abdominal wall assembly:   1. Cut a 20” by 24” piece of the following: faux suede/thin leather, quilt batting, muslin (cut two pieces), red felt, and beige swimsuit fabric 2. Using the tacky glue spray, glue two pieces of the cream felt side-by-side about 1.5” apart to the underside of the faux suede/thin leather piece. Set aside to dry. 3. Using the tacky glue spray, glue two pieces of the 9” x 12” red felt, side-by-side about 1.5” apart, to the large 20” x 24” piece. Set aside to dry. Once dry, cut the rectus diastasis. 4. Place the layers in the following order face down and staple them together: faux suede/thin leather 🡪 two pieces of muslin 🡪 red felt 🡪 beige swimsuit fabric. Set aside.   Assembly of uterus:   1. Cut a 12” x 23” piece from the exercise mat and using the hot glue, apply a piece of Velcro® to the top and bottom edges on the same side so that when folding the piece in half longwise, the Velcro® hold the ends together (this allows you to re-insert the amniotic sac for the second use) 2. Cut a 6” x 18” piece of tulle and fold it long ways in half. Using clear tape, tape the tulle to the bottom edge of the exercise mat. 3. Cut a 23” piece of saran wrap and using tape, attach it over the entire surface of the exercise mat.   Placenta and fetus:   1. Deflate the plastic ball and create a concave structure. Glue the loofah to the inside of this “bowl”. 2. Using the hot glue, glue a 14” piece of jumping rope to the convex surface of the plastic ball. Stitch the rope to the stuffed animal’s umbilicus or, if using the plastic baby doll, tape the jump rope to the baby doll’s umbilicus. 3. Place the placenta and baby doll inside the gallon plastic bag. For the updated version, fill the gallon bag with warm water. Place this bag inside the uterus.   Final assembly:   1. Fill the Sterilite® plastic box halfway with bubble wrap or plastic bags filled with stuffing. 2. Place the uterus containing the fetus inside the plastic box, and place the abdominal wall layers on top. 3. Using packing tape, tape all the side of the abdominal wall to the plastic box. 4. Note, the plastic box handle on either side acts as the pubic symphysis. | 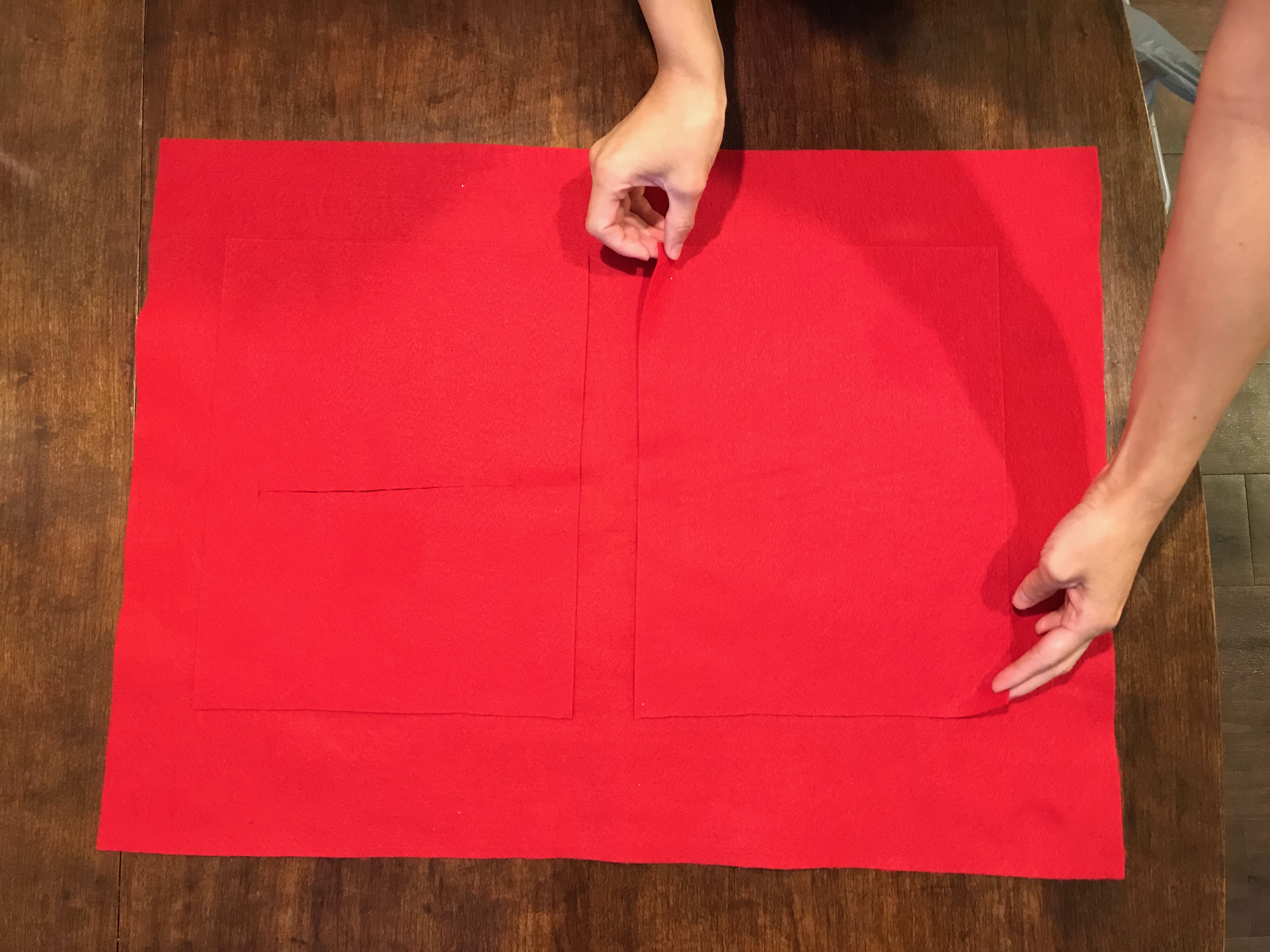  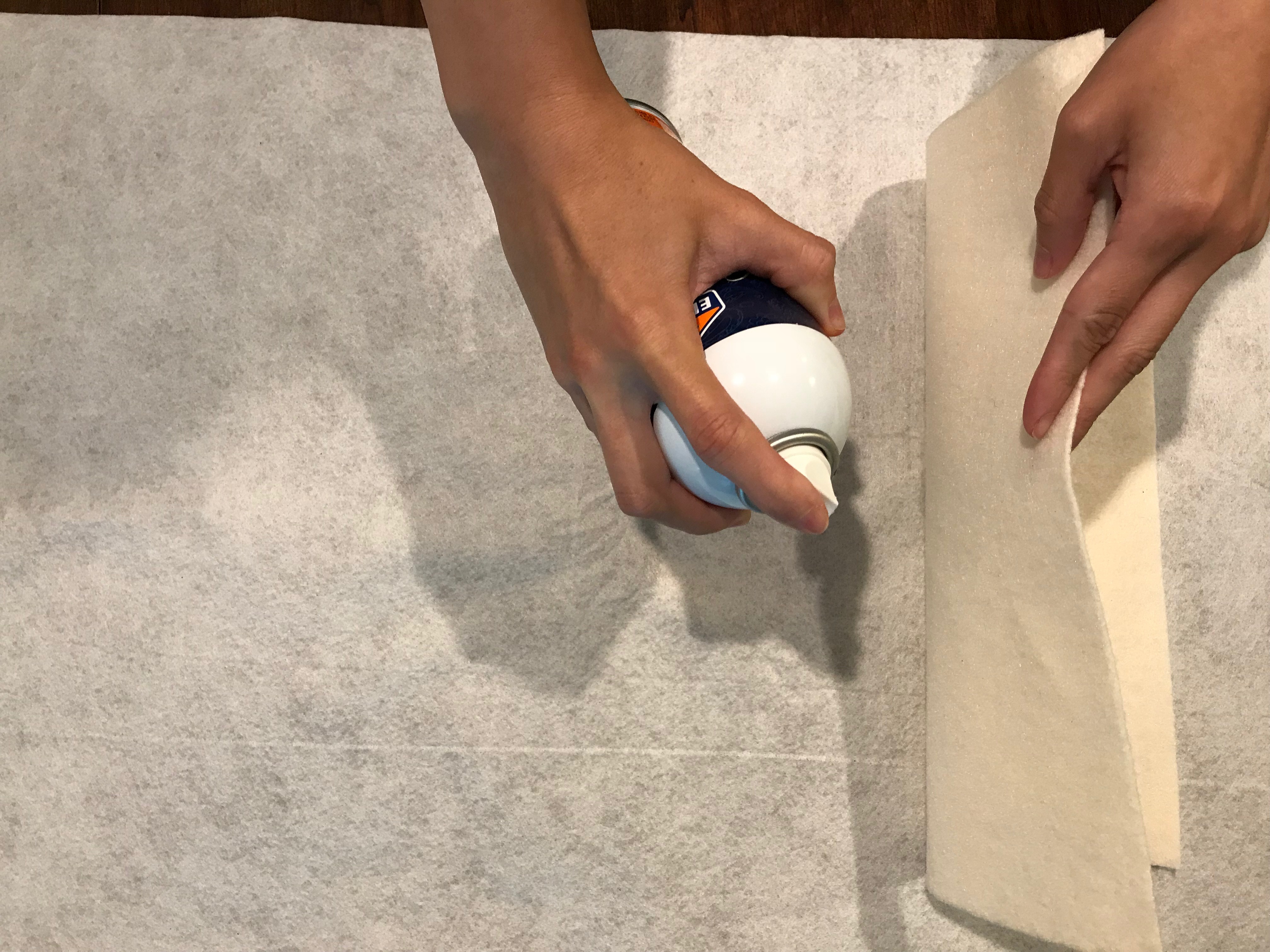  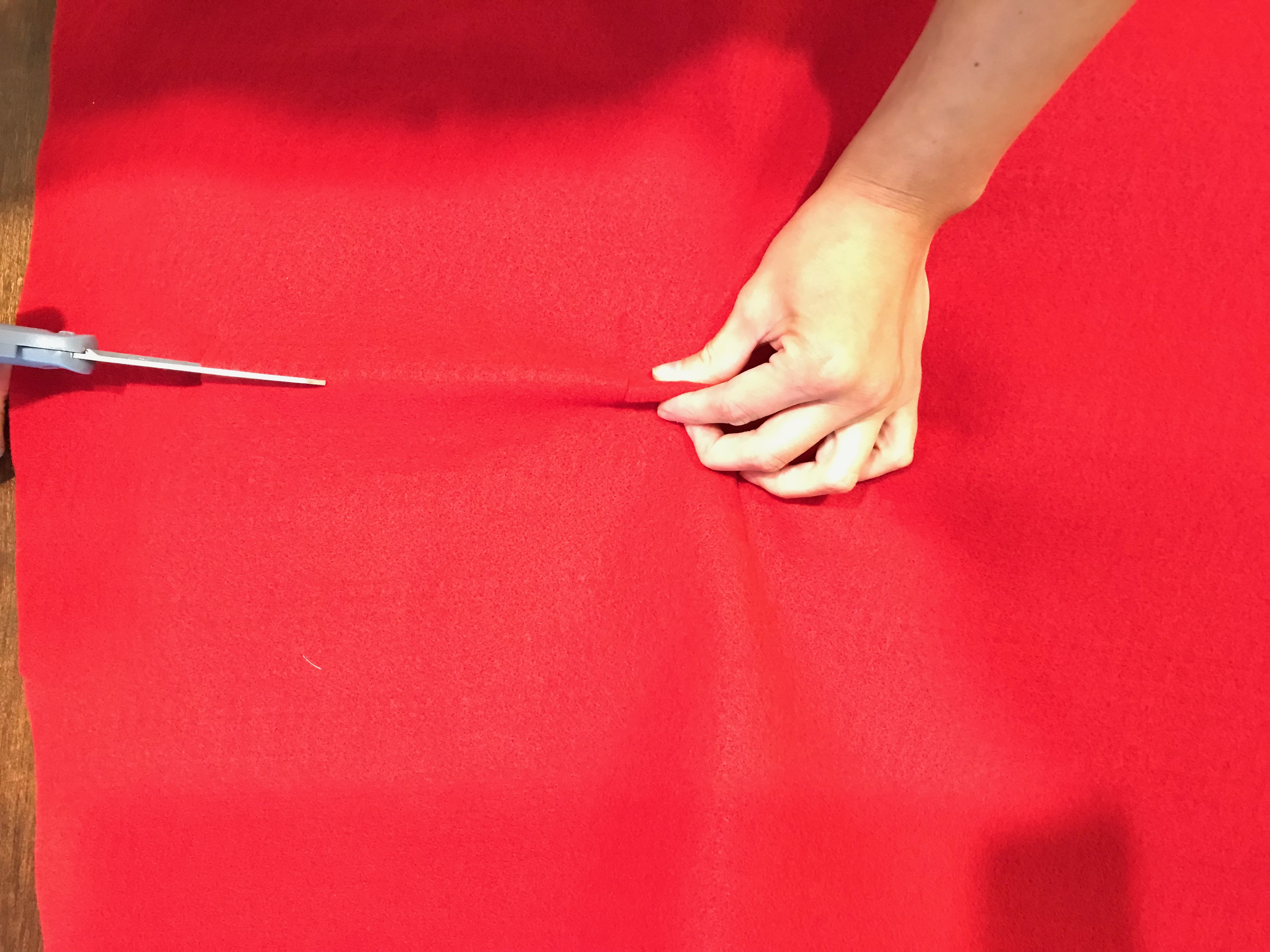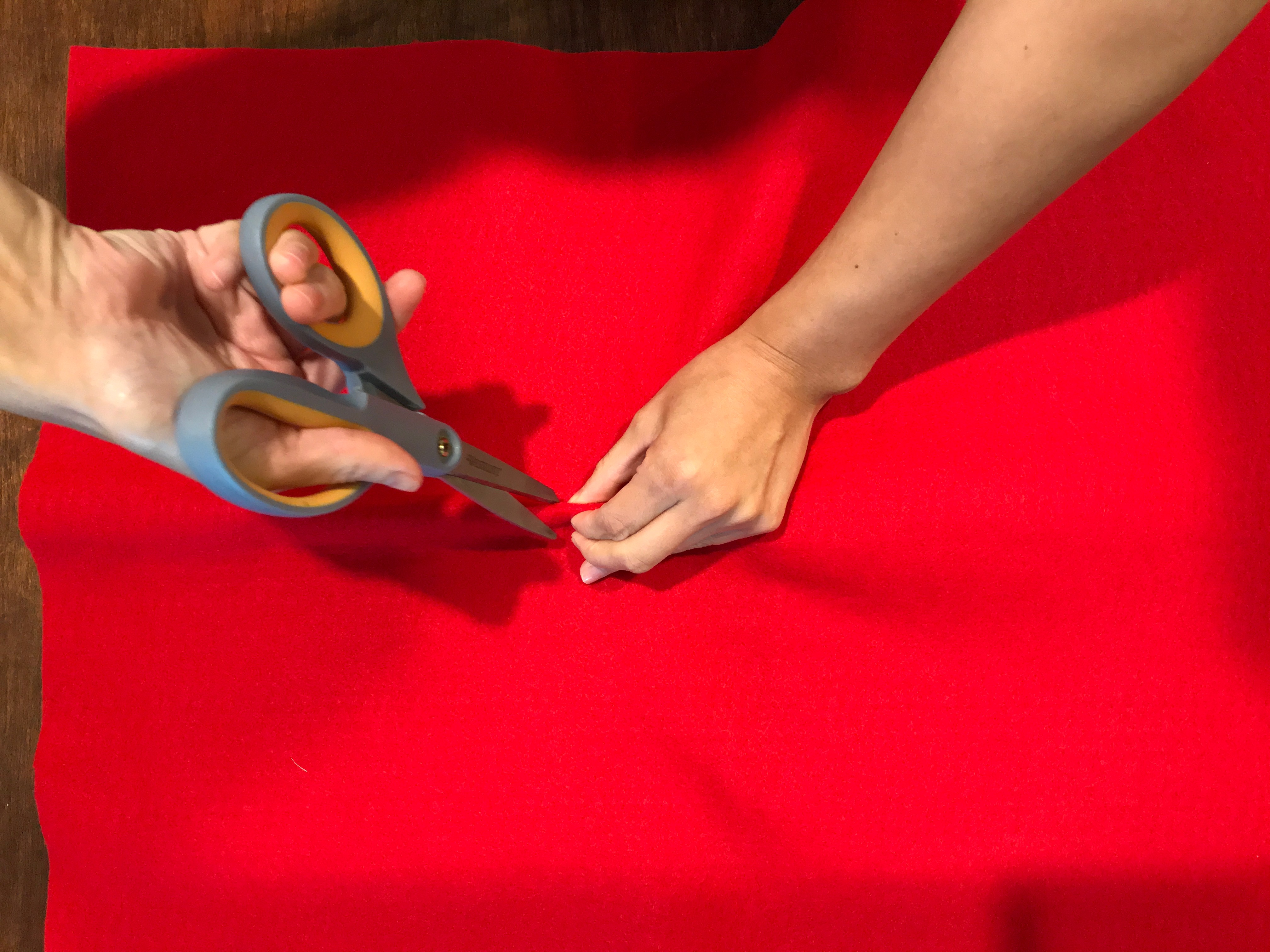  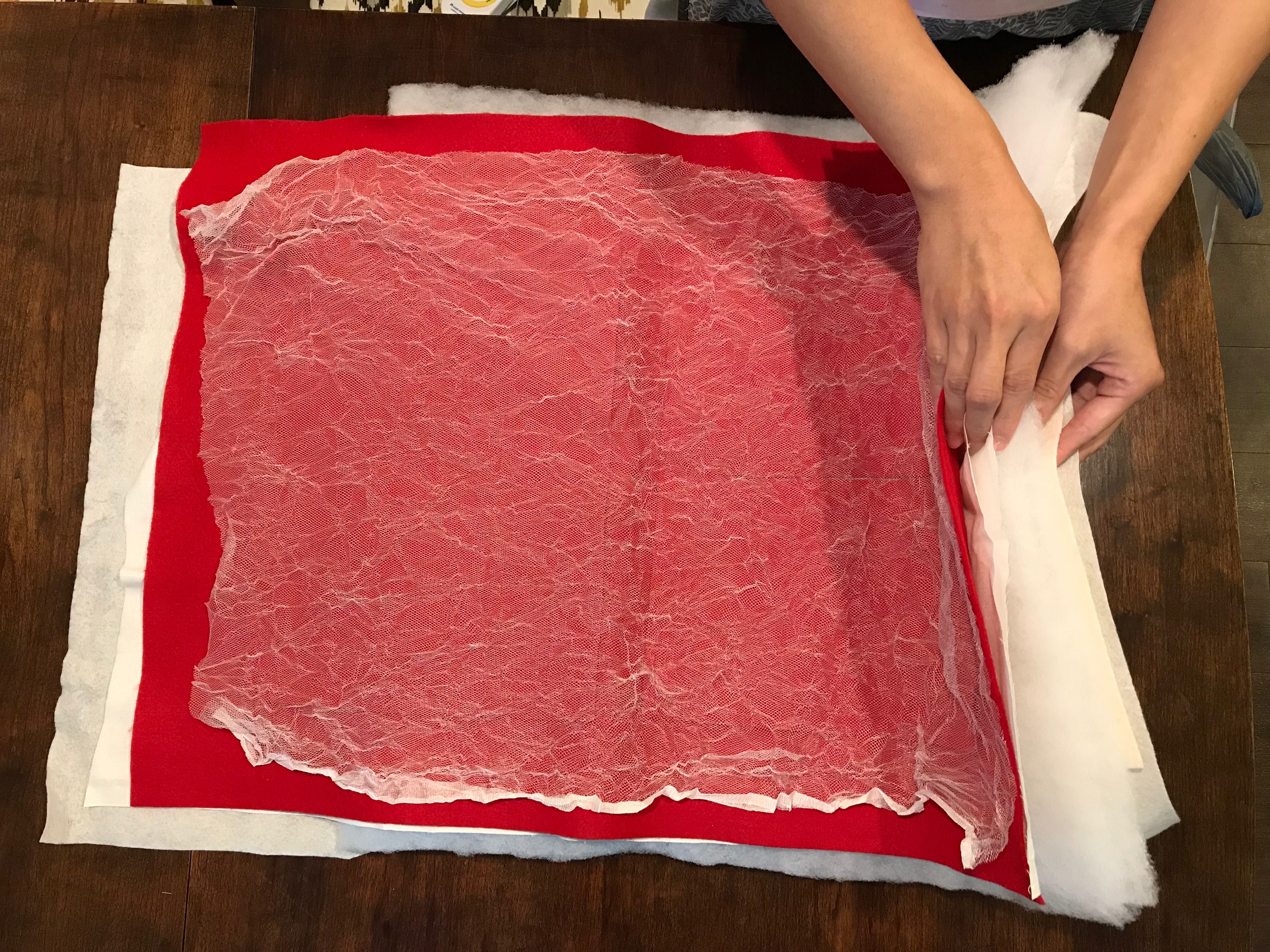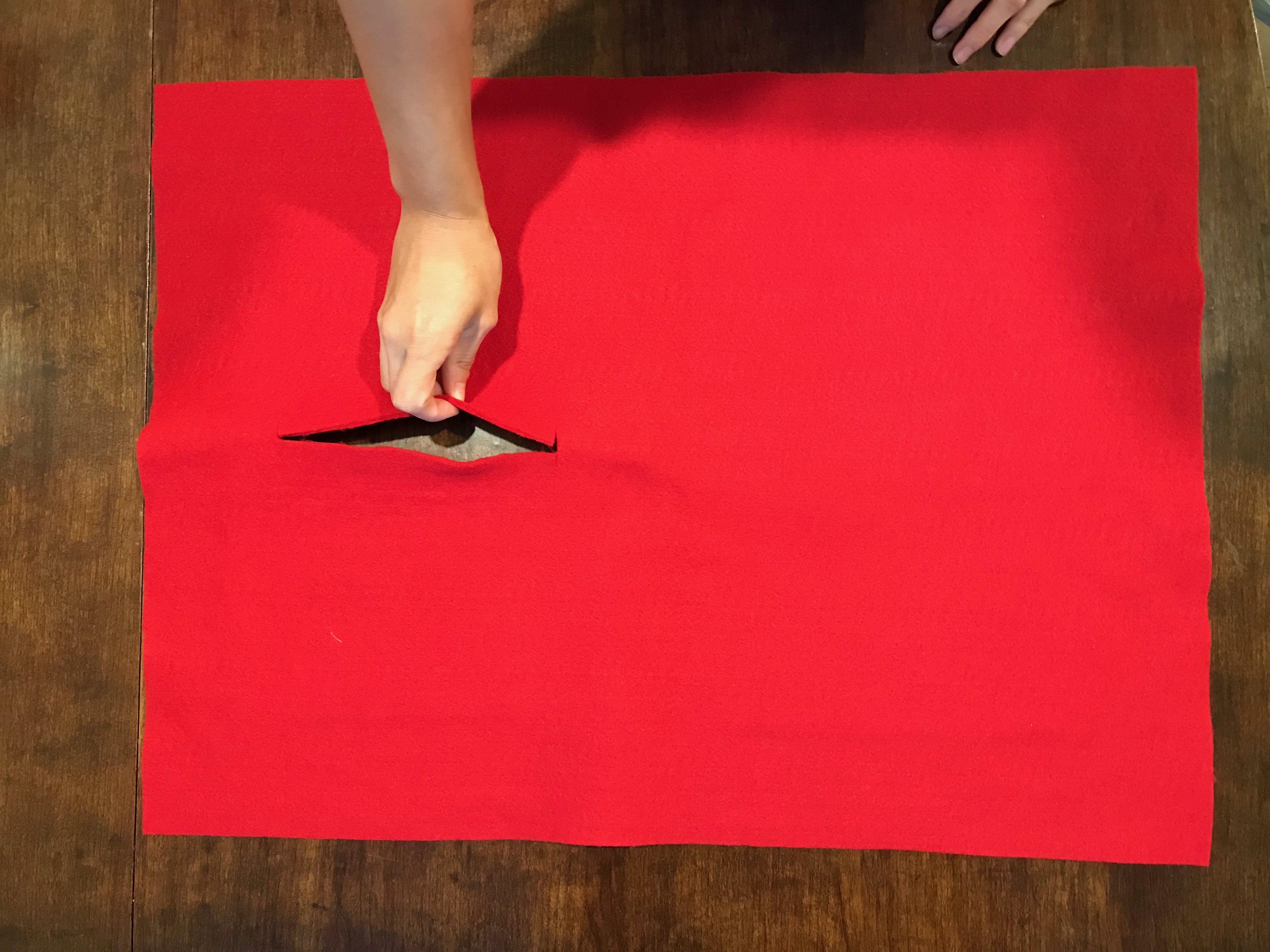  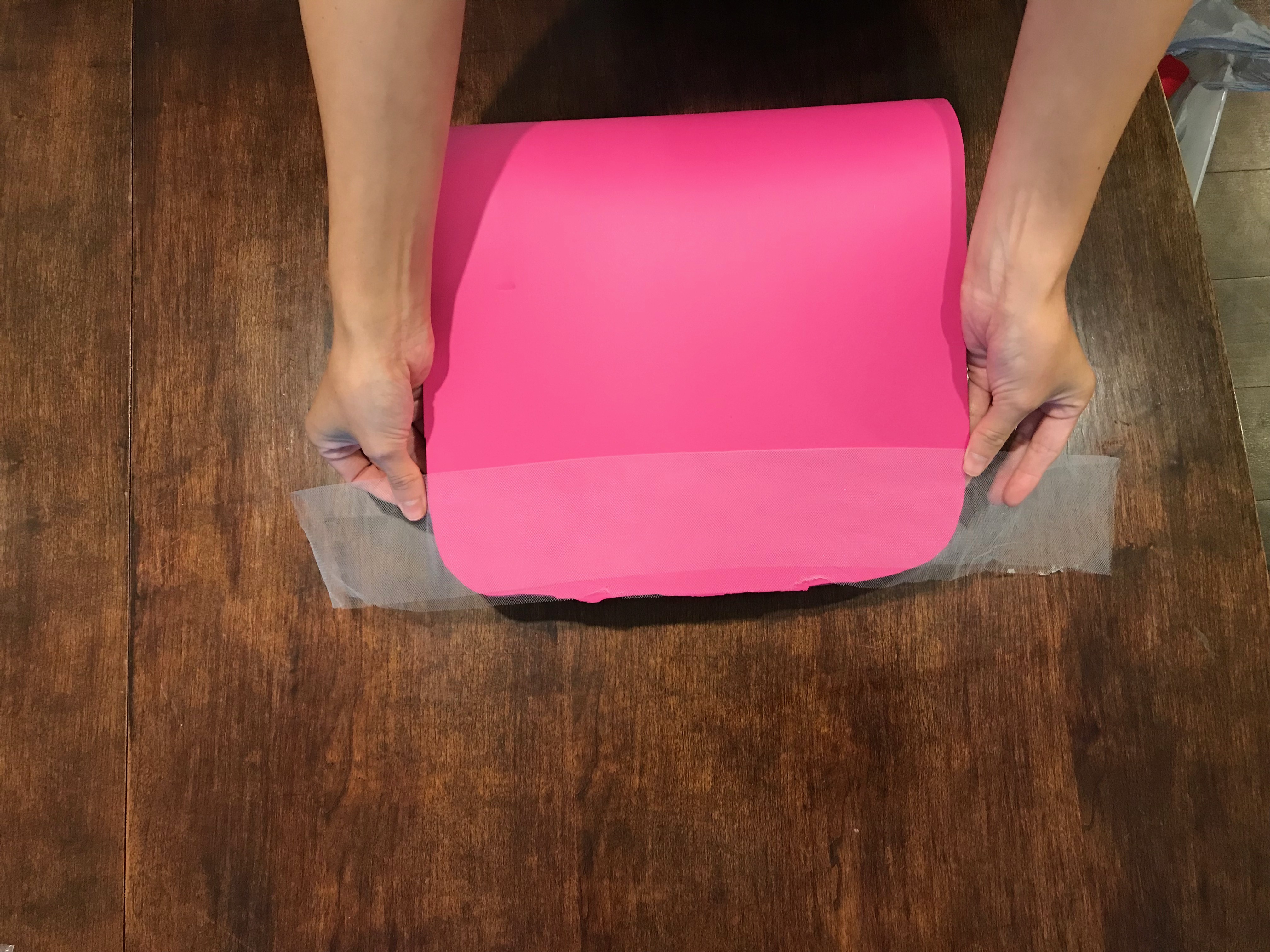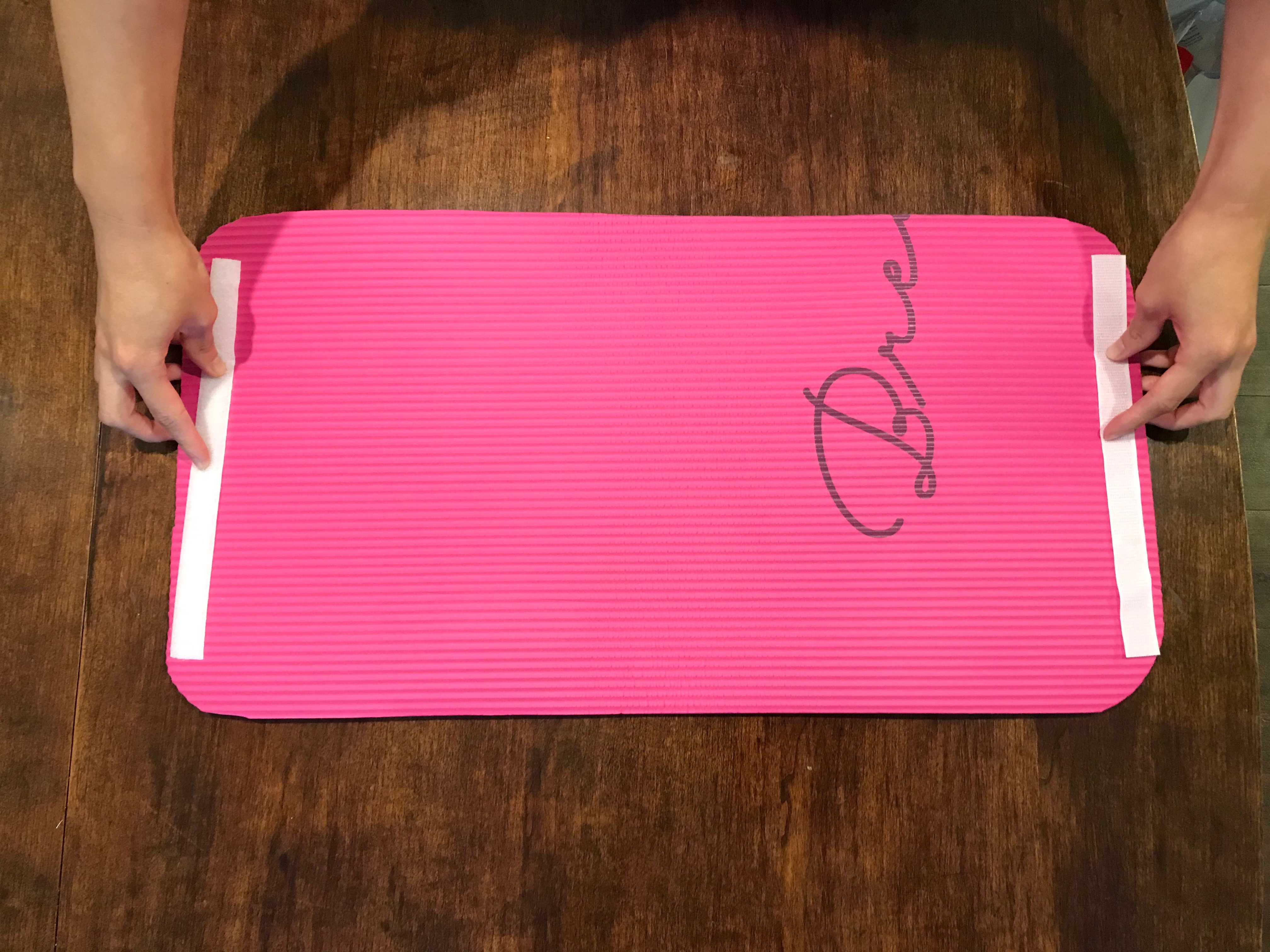  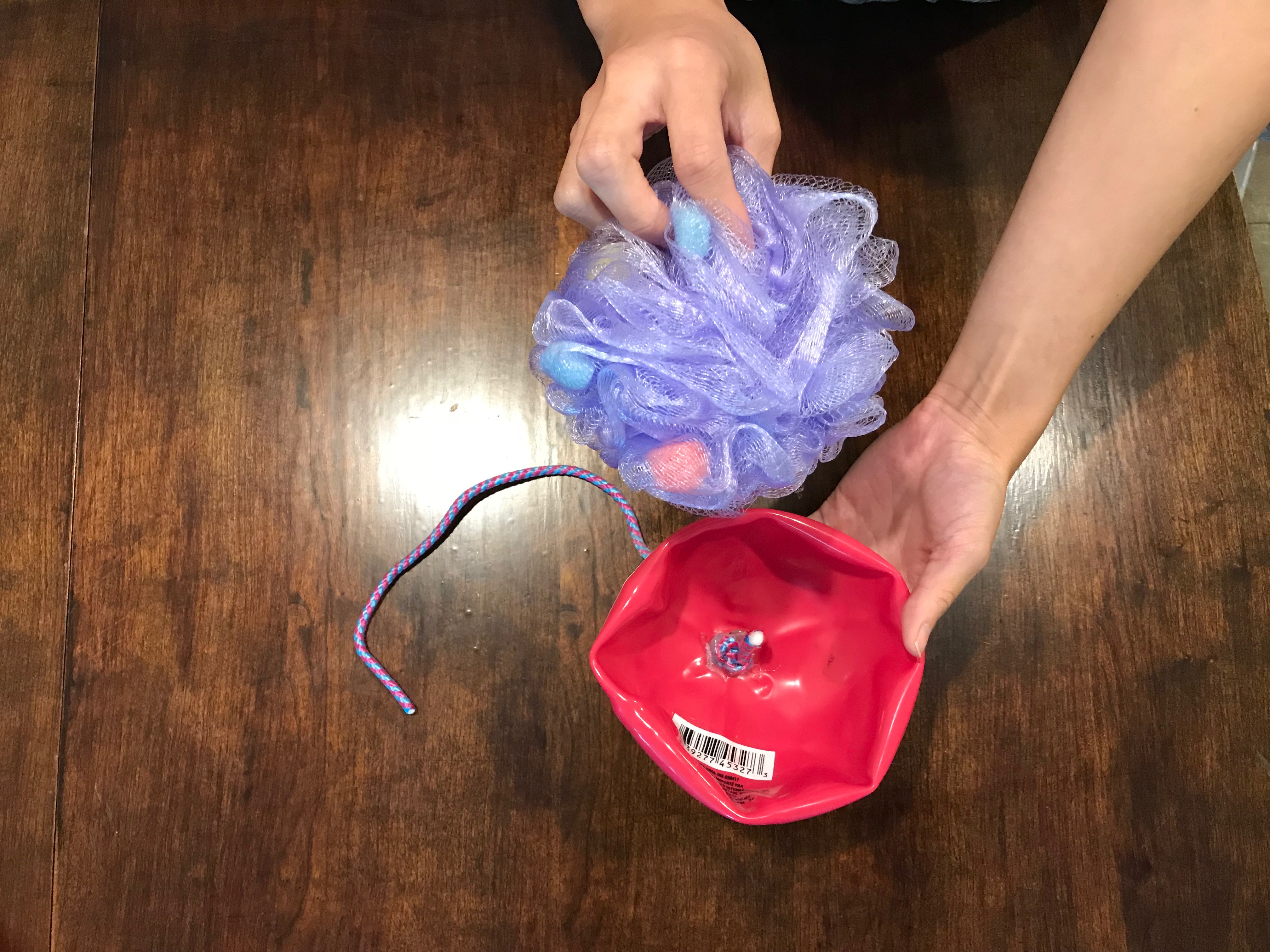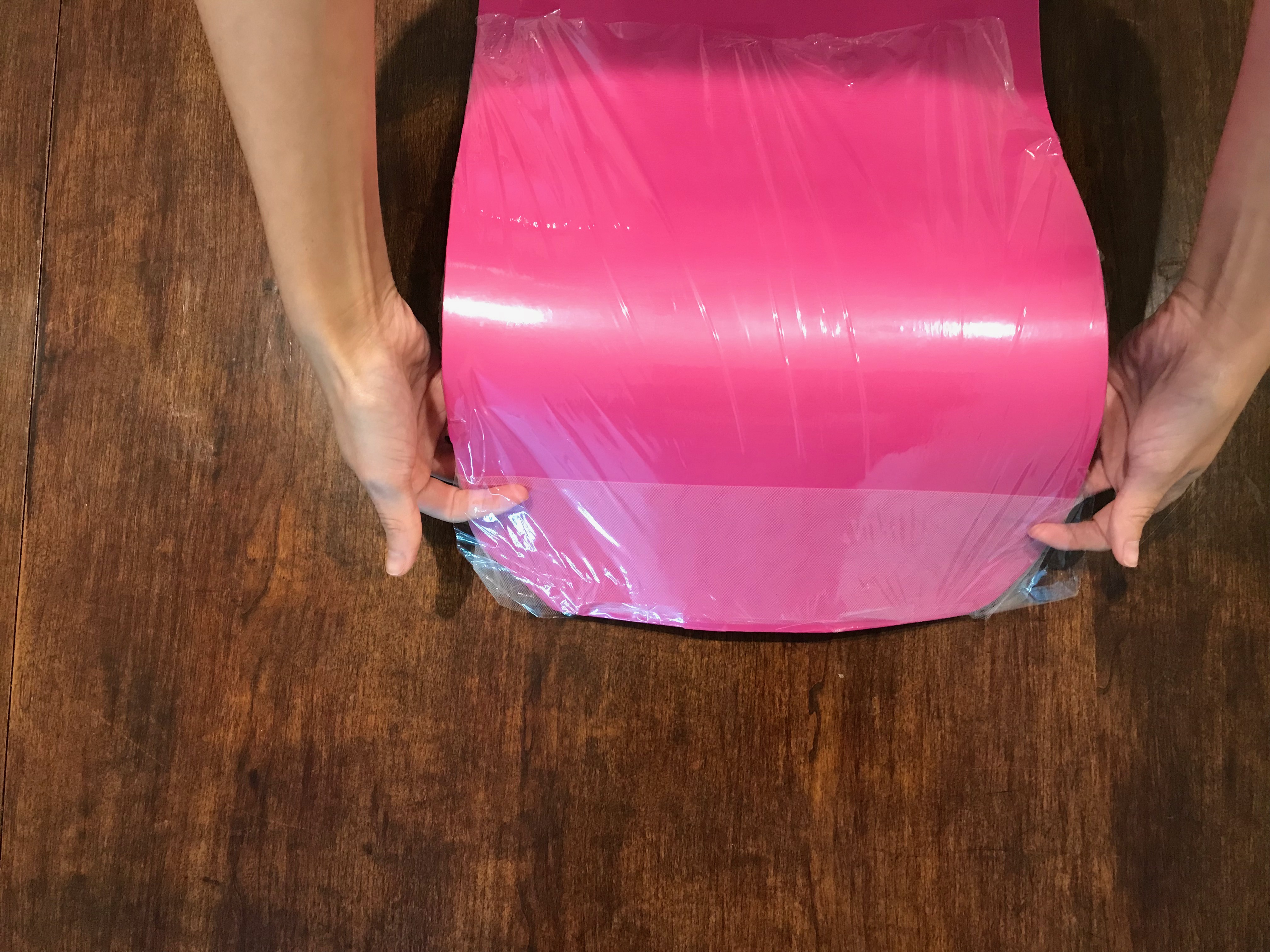  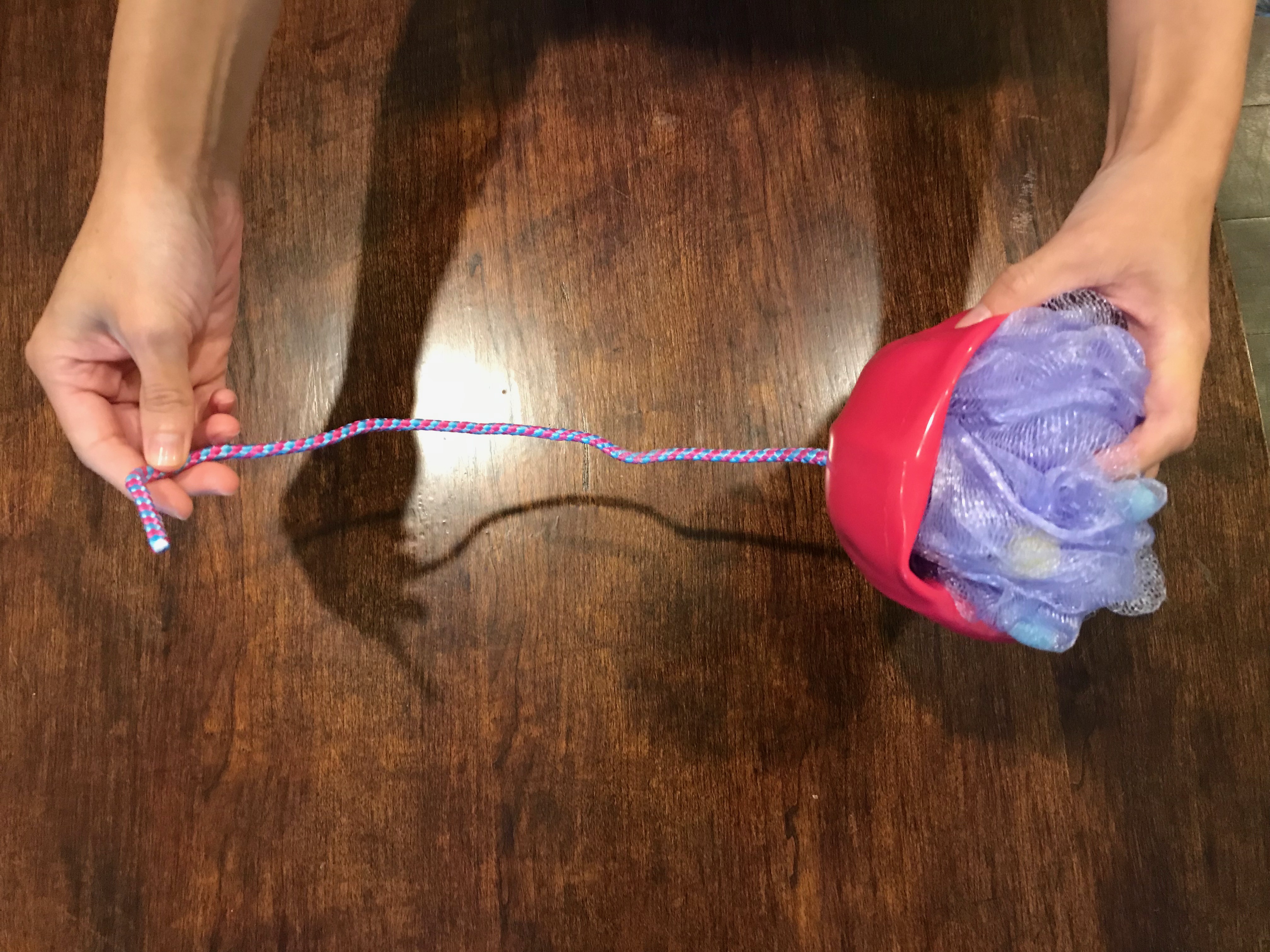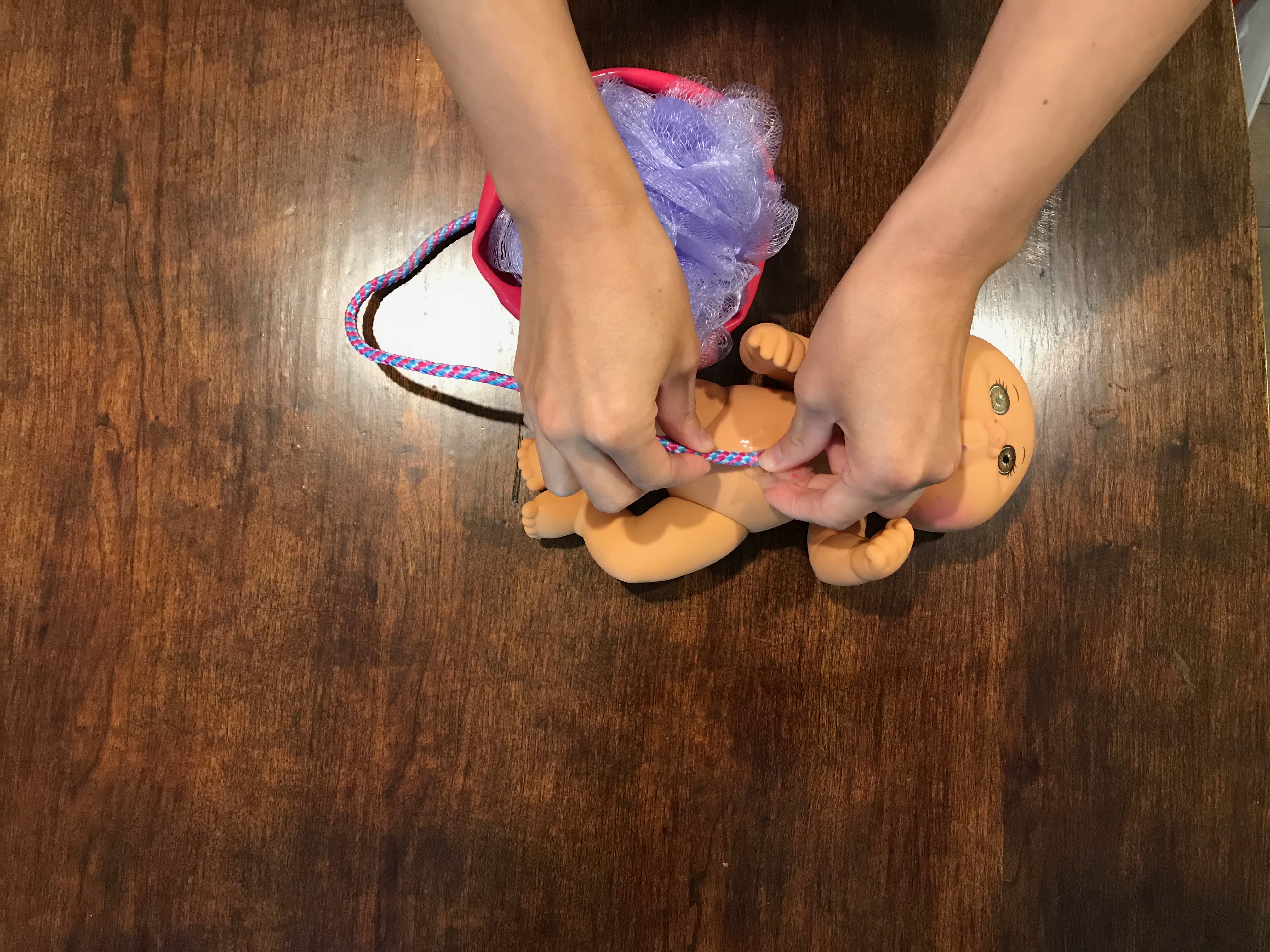  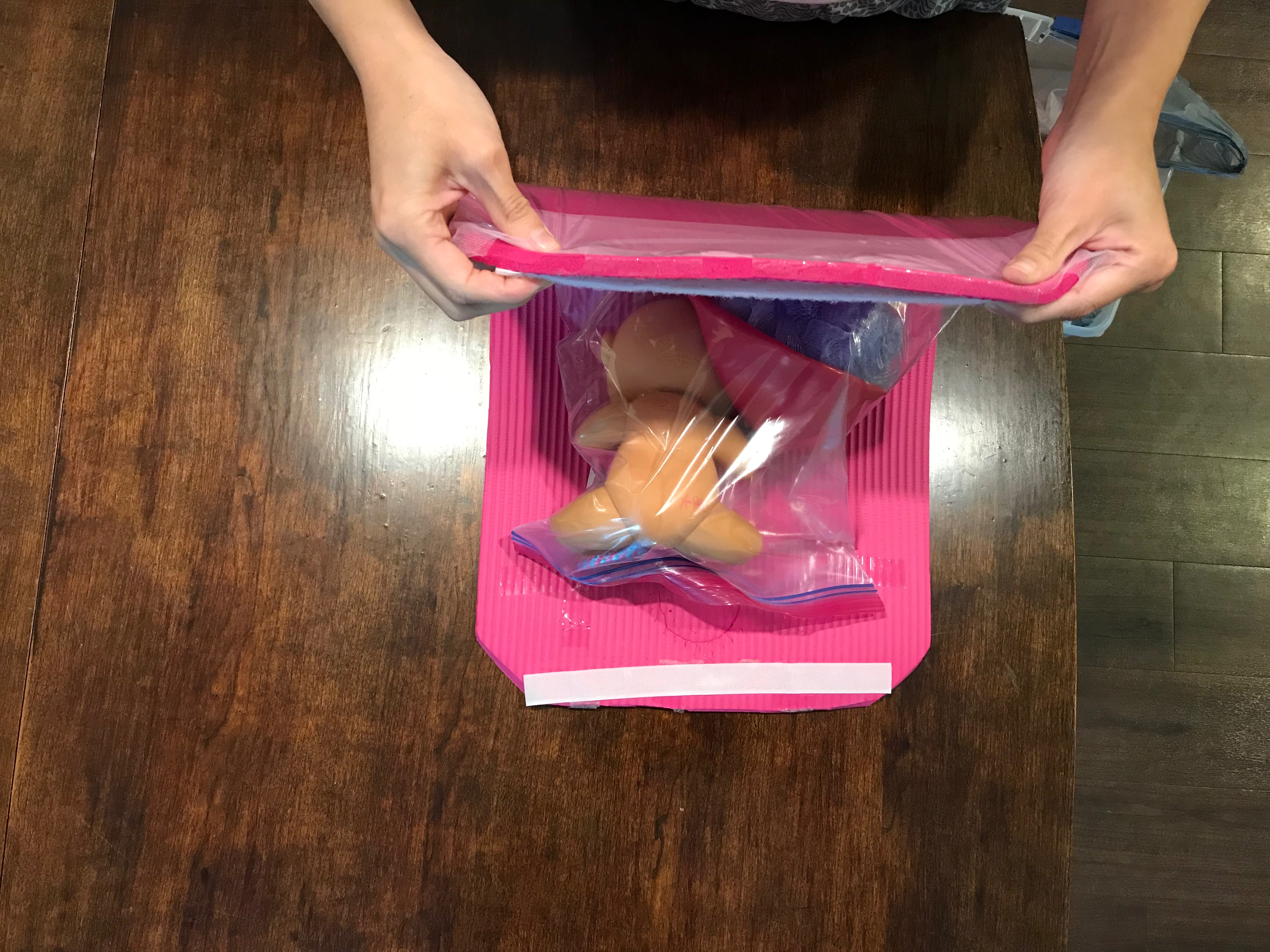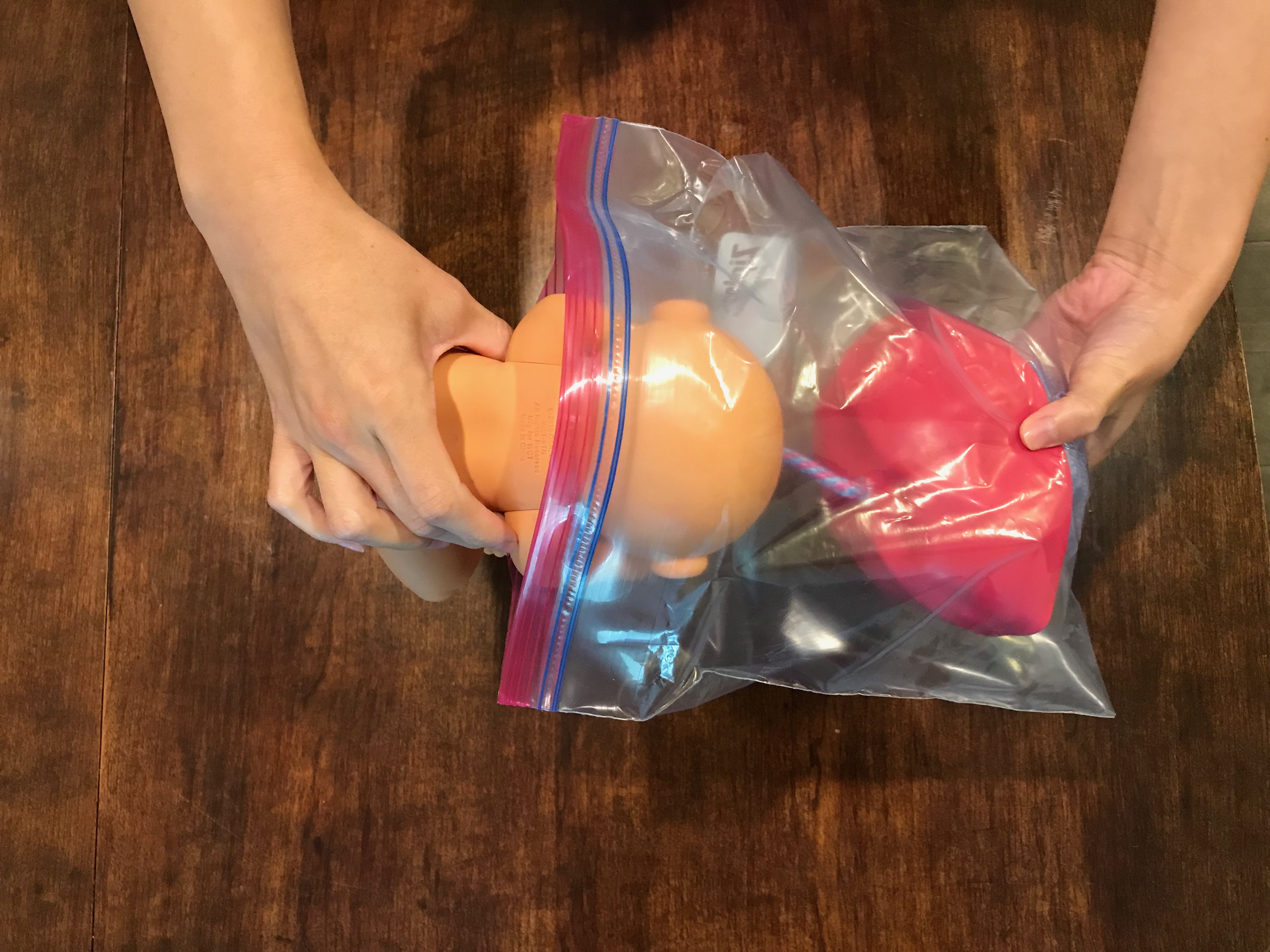  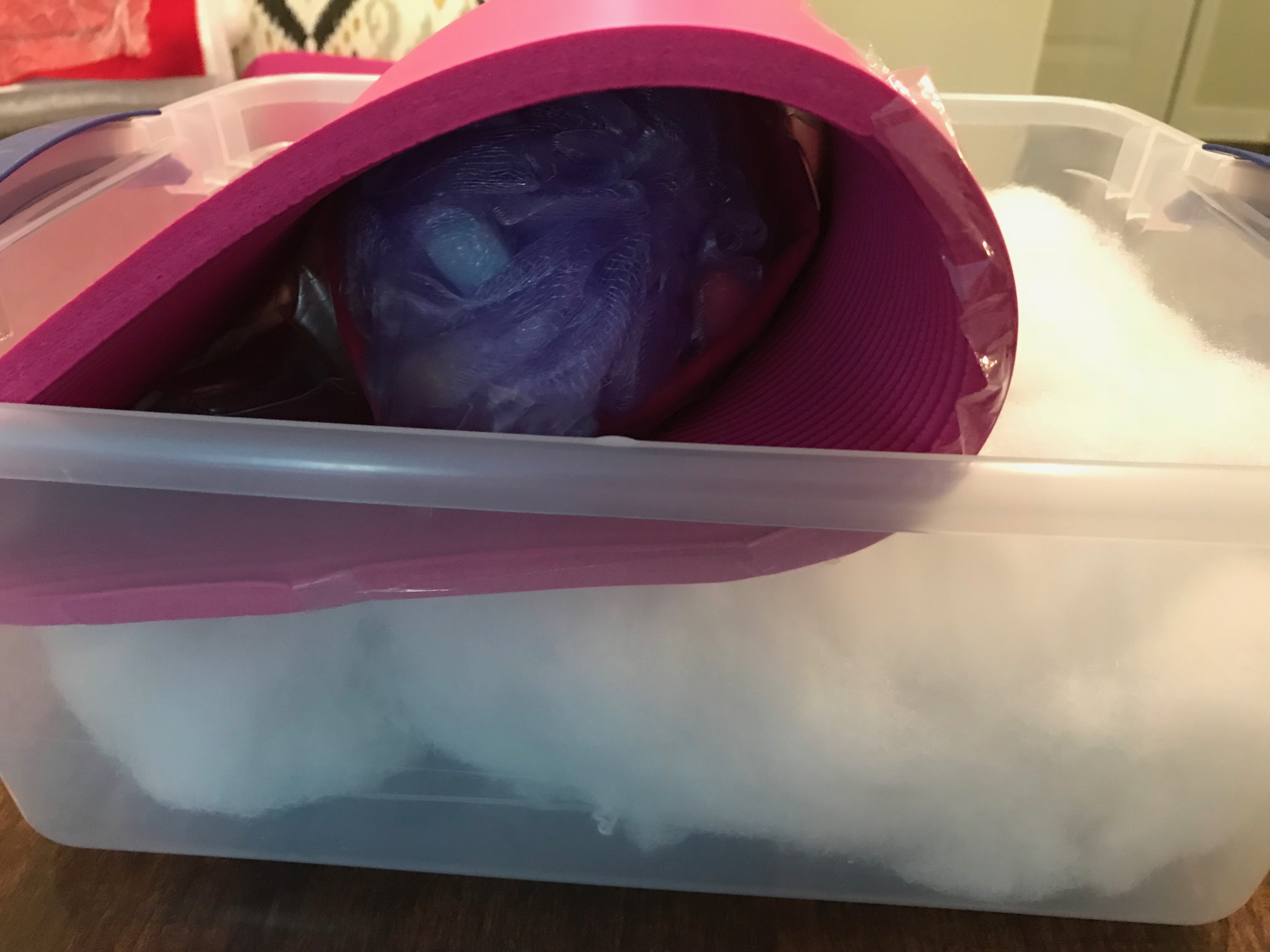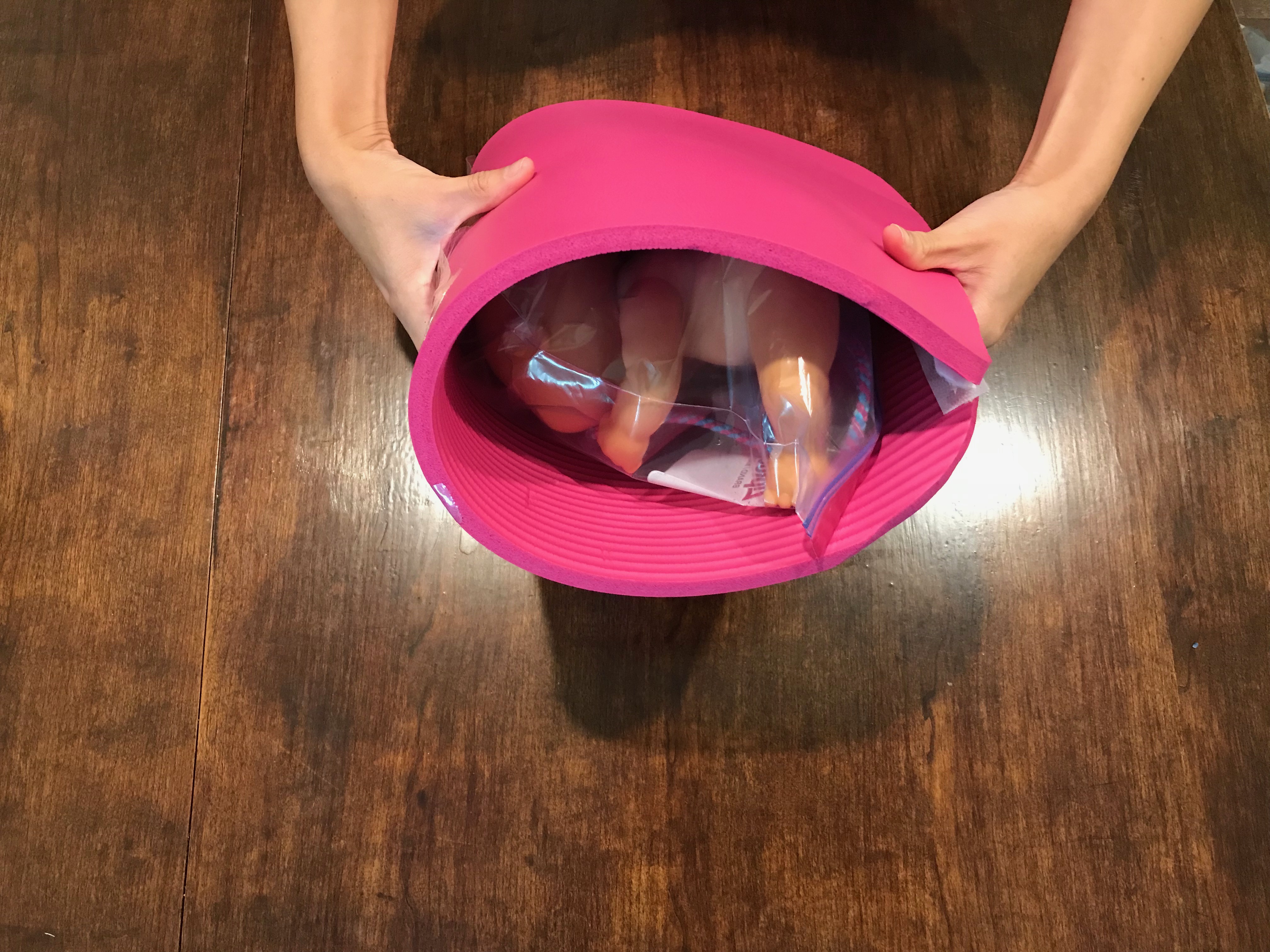  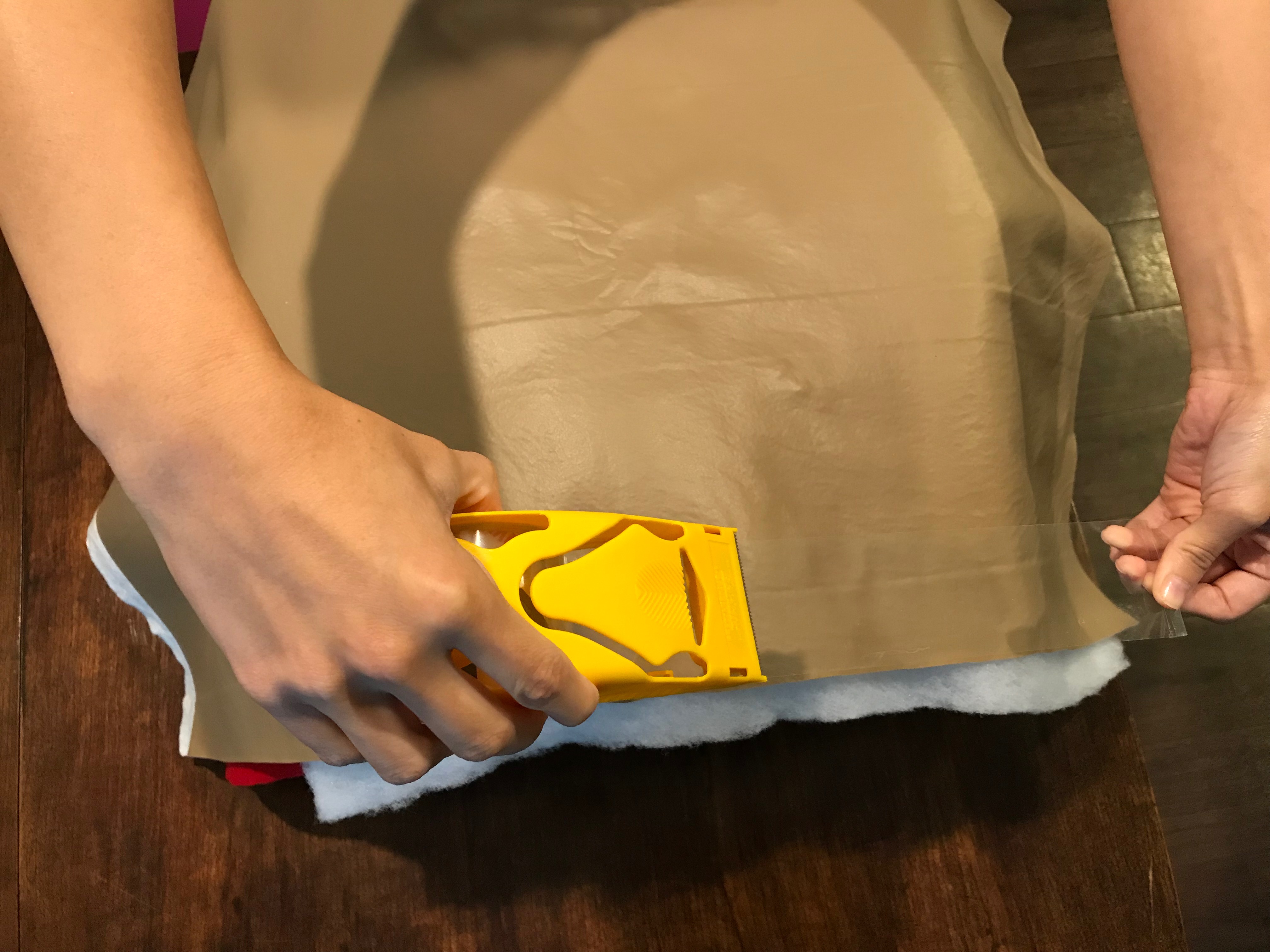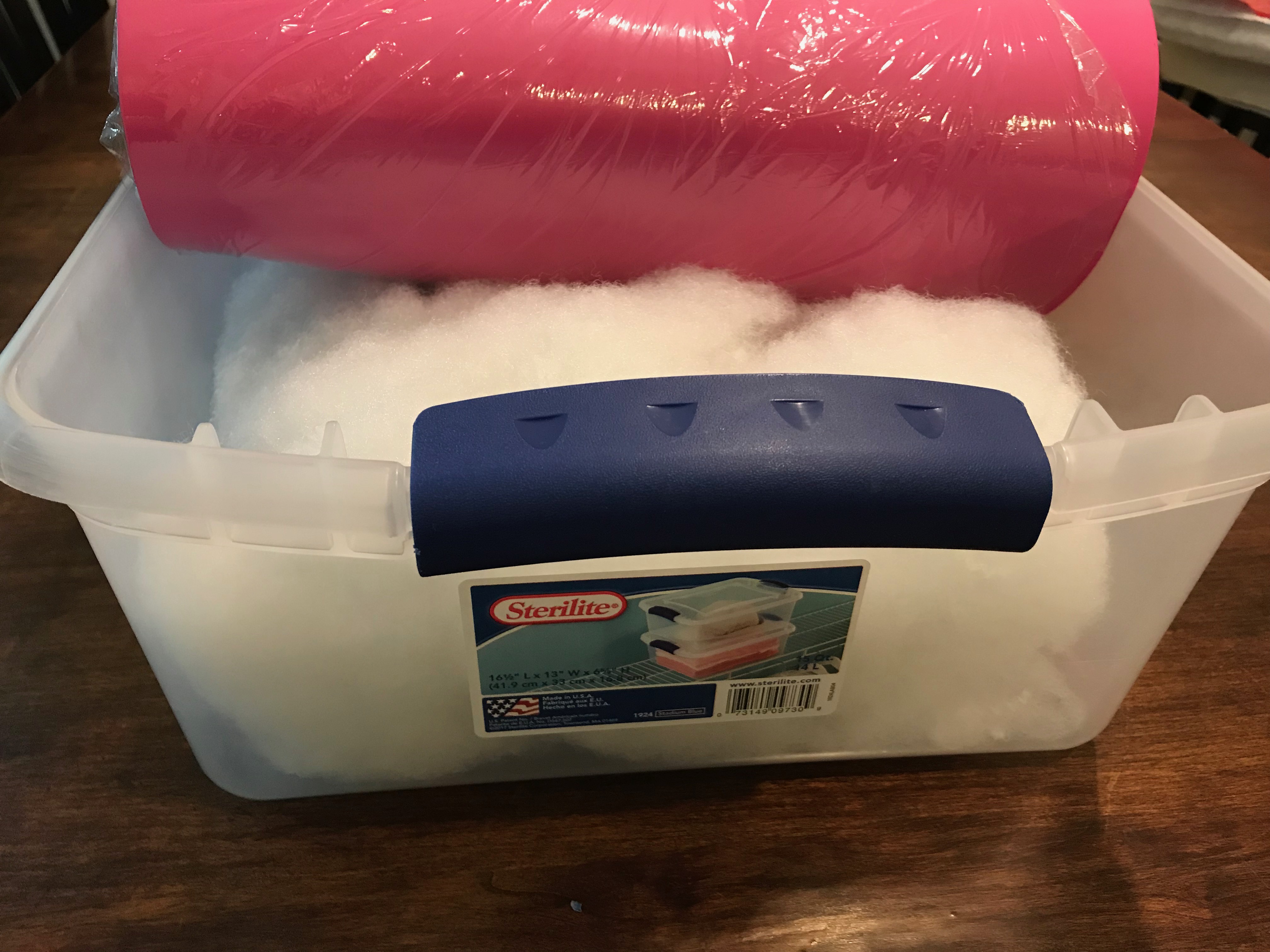  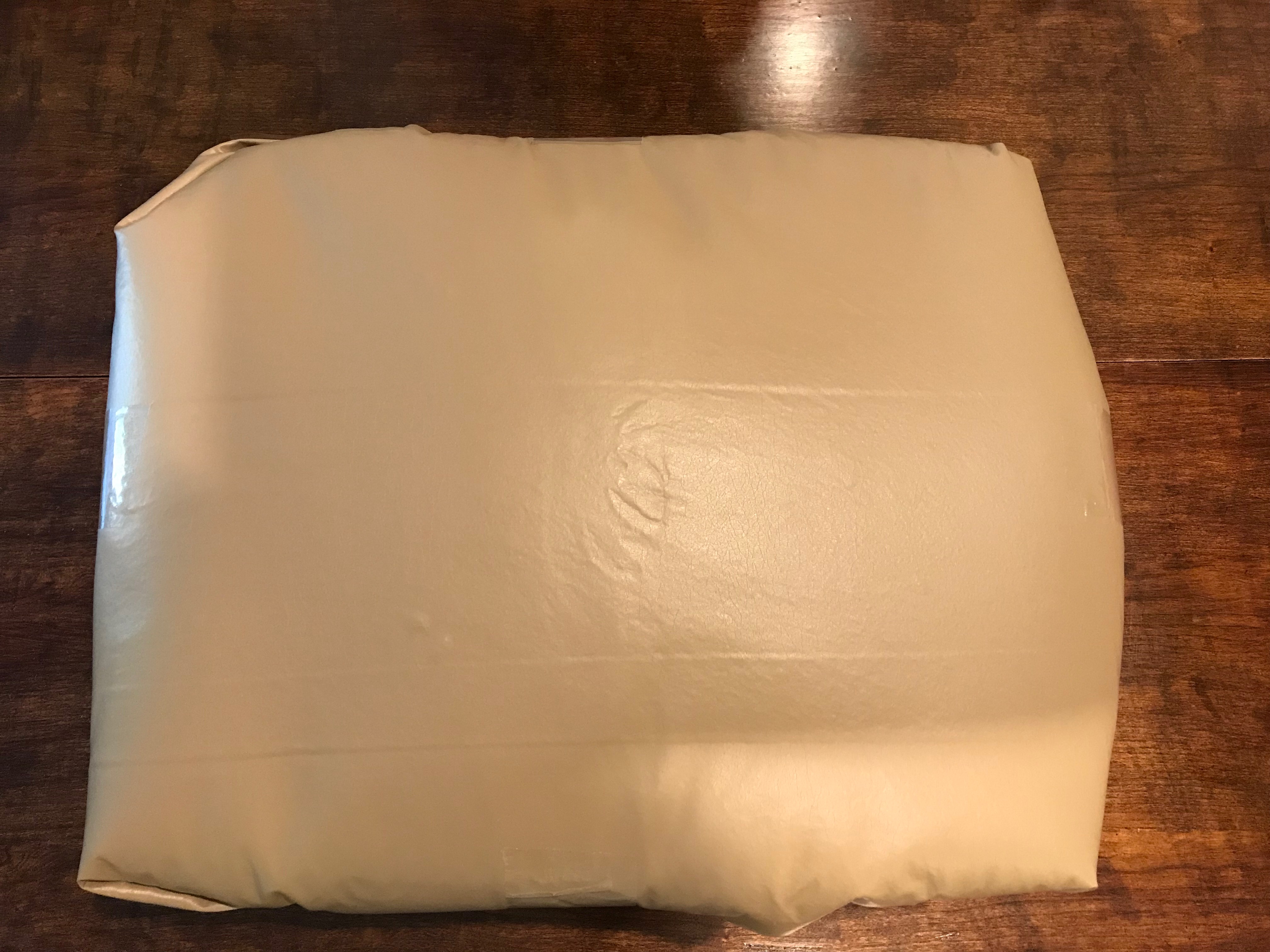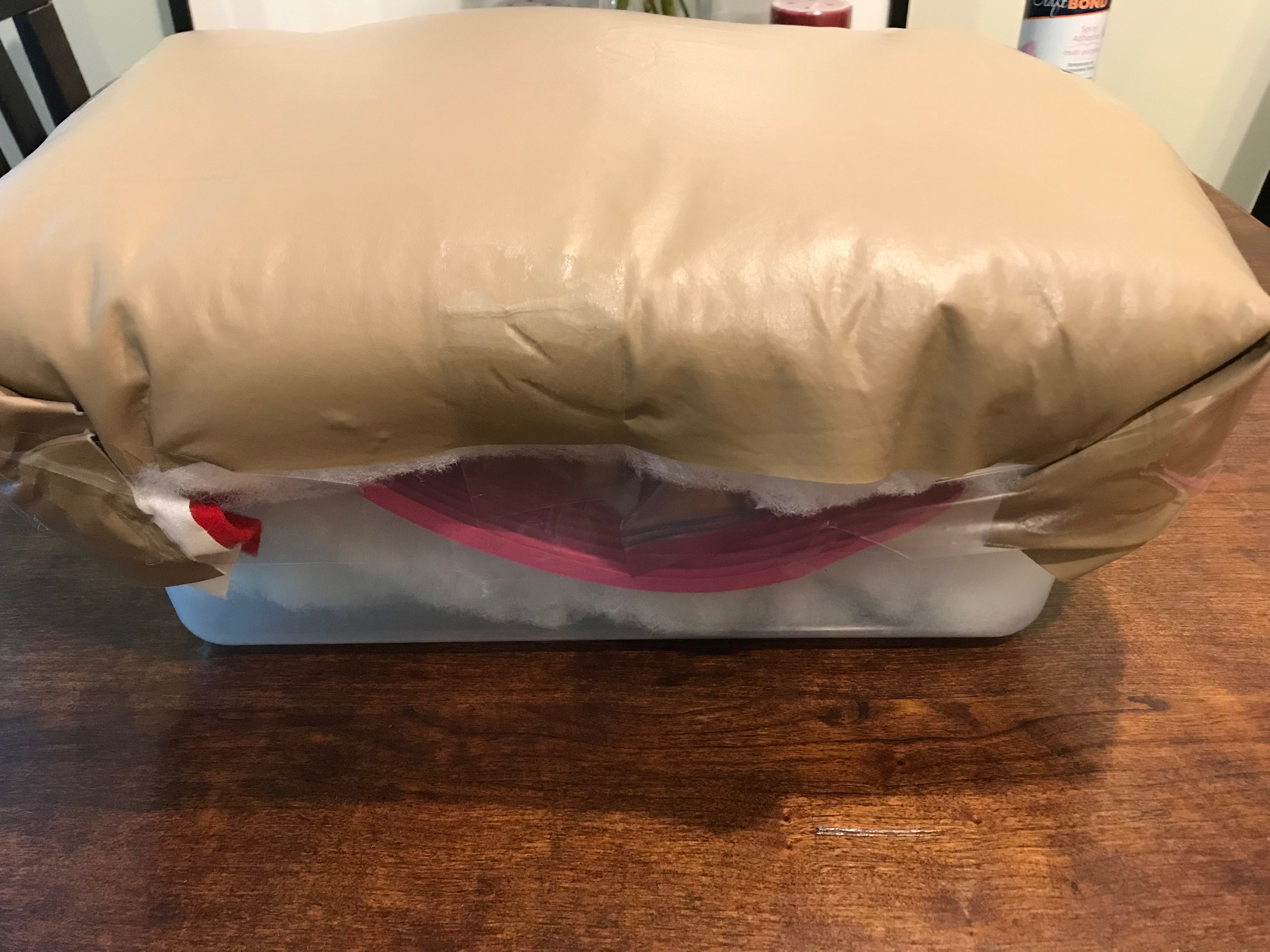 |
|  | Two pieces of 9” x 12” cream felt (subcuticular layer) | $0.23/piece ($0.46) |  |  |
|  | Tacky glue spray | $7.99 ($0.80) |  |  |
|  | Quilt batting (Subcutaneous tissue) | $4.97/1.53 yards ($0.99) |  |  |
|  | Muslin (Two layers of rectus fascia) | $4.99/yard ($1.66) |  |  |
|  | Red felt 17.8” x1 7.8 “(Rectus abdominis) | $0.99/piece ($0.99) |  |  |
|  | Two pieces of 9” x 12” red felt (Rectus abdominis) | $0.23/piece ($0.46) |  |  |
|  | Beige swimsuit fabric (Peritoneum) | $4.72/yard ($0.59) |  |  |
| Uterus and Placenta | Exercise mat, pink/red, ½ inch extra thick (uterus) | $19.99/mat ($3.33) |  |  |
|  | Tulle (bladder) | $2.99/yard ($0.12) |  |  |
|  | Saran wrap (vesicouterine peritonium) | $1.66/ 200 ft^2^ roll ($0.02) |  |  |
|  | Shower loofah (cotyledons) | $1/loofah ($1) |  |  |
|  | 5” diameter plastic ball (placenta) | $1/ball ($1) |  |  |
|  | Jumping rope (umbilical cord) | $1/rope ($0.25) |  |  |
|  | 1 Gallon Ziploc Bag  (amniotic sac) | $4.78/box ($0.13) |  |  |
| General supplies | 1 Pair of fabric scissors | Used existing |  |  |
|  | 1 Roll of tape | $0.97/roll ($0.10) |  |  |
|  | 1 Hot glue gun and sticks | *Used existing* |  |  |
|  | 1 Roll of packing tape | $3.34/roll ($0.33) |  |  |
|  | 1 Roll of bubble wrap | $5.44/roll ($5.44) |  |  |
|  | 1 packet of Velcro® squares | $4.27 ($2.12) |  |  |
|  | Stapler | *Used existing* |  |  |
|  | Sterilite® plastic box 16.5”L x 13”W x 6.625”H with handles | $3.97/box ($3.97) |  |  |
|  | 1 roll of Velcro® | $0.99 per roll ($0.49) |  |  |
|  | 10” long, all-plastic baby doll with flexible limbs (if implementing amniotic fluid) | $8.86/doll ($8.86) |  |  |
|  | Stuffed animal (if not implementing amniotic fluid) | *Used existing* |  |  |

**Total Price ~ $25**

**Total Price with plastic doll ~ $34**

The list below includes the supplies that are needed to run each high-fidelity simulation. Items with the astirix (*) represent the minimum necessary materials.

- One mannequin torso with head (no pelvis)
- C-section model already assembled*
- One IV pole, one bag of IV fluid, and IV tubing
- One table (rectangular is ideal) – simulates the table in the operating room*
- Instrument table* and Mayo stand
- One Cesarean section tray*
- Suture material per standard fashion for Cesarean section (this includes one suture for closing each of the following layers/structures: uterus, fascial, subcutaneous tissue, and skin)*
- Bovie disposable electrosurgical rocker pencil
- Suction tip and tubing
- One skin prep/cleansing kit
- Cesarean section surgical drape
- Operating room attire: hats, face shields or masks/goggles, surgical gowns, shoe coverings
- Surgical gloves ^[[1]](#footnote-1)^
- Tablet or iPad and access to a projector

1. We strongly recommend using sterile surgical gloves, if possible. This much better simulates operating, as the learners will never operate barehanded. If surgical gloves are not available due to cost, close fitting exam gloves can easily be substituted. [↑](#footnote-ref-1)
