# Supplementary material for: Improving Learners' Comfort With Cesarean Sections Through the Use of High-Fidelity, Low-Cost Simulation
Source: MedEdPORTAL. 2020 Feb 14;16:10878. doi: 10.15766/mep_2374-8265.10878 (PMC7062555; doi:10.15766/mep_2374-8265.10878)
Supplement: Supplementary file 1 — A. Simulation Case.docx B. CS Model Assembly and Materials.docx C. Surgical Instruments.pptx D. CS Steps and Time-out.docx E. Presimulation Survey.docx F. Postsimulation Survey.docx G. Simulation Images.docx H. Critical Actions Checklist.docx I. Debriefing Materials.docx [file mep-16-10878-s001.zip › D. CS Steps and Time-out.docx]

**Appendix D:** Cesarean Section Steps, Instrument Call and Surgical Time-out

| Time-Out Steps* | Simulation |
| --- | --- |
| 1. Patient identification 2. Consent for procedure 3. Consent for blood 4. Allergies 5. Antibiotics 6. Position 7. X-rays and implants 8. Electrosurgery level 9. Planned specimens 10. Agreement | 1. This is Amy Nitis, date of birth 05/12/1985, MRN 173856 2. She is consented for C-section, declines BTL 3. She has a consent for blood 4. She has no known allergies 5. Ampicillin, Gentamicin, and Clindamycin given 6. She is in the dorsal supine position with a leftward tilt 7. No x-rays or implants 8. Bovie set to 50/50 9. Planned specimens: placenta to pathology 10. All in agreement? |

| Call for… | …in order to |
| --- | --- |
| 1. Allis clamp | Test level of anesthesia |
| 2. Knife with #10 blade | Make first skin incision: Pfannenstiel incision |
| 3. Two Richardson retractors, a pair of Ferris-Smiths, and Mayos (curved) | Dissect the fascia |
| 4. Two Kocher clamps | Lift up the fascia |
| 5. Mayos (curved) | Dissect the fascia off the rectus abdominis muscle and bluntly separate the rectus muscle |
| 6. Two Kelly clamps and Metzenbaum scissors | Grasp and dissect the peritoneum and bluntly extend the dissection |
| 7. Richardson retractor and bladder blade | Retract back the abdominal wall and protect the bladder |
| 8. Russian forceps and Metzenbaum scissors | Dissect the vesicouterine lining to create a bladder flap |
| 9. Knife with #10 blade | Make a low transverse hysterotomy incision and suction as you cut |
| 10. Allis clamp | Rupture the amniotic sac |
| 11. (Remove bladder blade) | Deliver using standard breech maneuvers |
| 12. Plastic clamps and scissors | Clamp and cut the cord (get ready to remove the placenta via spontaneous, expressed, or manual extraction) |
| 13. Pennington clamp | Remove trailing membranes (if needed) after removing the placenta |
| 14. Dry lap sponge | Clear out uterus of all debris |
| 15. Russians and 0-Monocryl (then suture scissors when ready) | Close uterine incision using running locked suture technique. Inspect for hemostasis. |
| 16. Two hemostats | Tag suture tails (no need to tag if uterus is not externalized) |
| 17. Two Kocher clamps | Inspect for hemostasis and then inspect the subfascial spaces |
| 18. Two Richardson, Ferris-Smiths, and 0 or 1.0 PDS (then suture scissors) | Close the fascia using running suture technique |
| 19. Bovie | Inspect the subcutaneous spaces for hemostasis. If bleeding, use electrosurgery. If patient is obese, close the adipose layer via interrupted sutures (may use 3.0 Vicryl or Monocryl) |
| 20. Adsons and 4.0 Vicryl on KS needle (then suture scissors) | Close the skin in a subcuticular fashion |

*The steps of the surgical time-out may vary by institution
