# Supplementary material for: Improving Learners' Comfort With Cesarean Sections Through the Use of High-Fidelity, Low-Cost Simulation
Source: MedEdPORTAL. 2020 Feb 14;16:10878. doi: 10.15766/mep_2374-8265.10878 (PMC7062555; doi:10.15766/mep_2374-8265.10878)
Supplement: Supplementary file 1 — A. Simulation Case.docx B. CS Model Assembly and Materials.docx C. Surgical Instruments.pptx D. CS Steps and Time-out.docx E. Presimulation Survey.docx F. Postsimulation Survey.docx G. Simulation Images.docx H. Critical Actions Checklist.docx I. Debriefing Materials.docx [file mep-16-10878-s001.zip › F. Postsimulation Survey.docx]

**Appendix F:** Post-simulation survey

Please circle your level below:

PYG1 PGY2 PGY3 PGY4 M3 Other__________________

Using the following scale please answer the questions below:

1= Poor 2 = Fair 3 = Good 4 = Very Good 5 = Excellent

1. What is your current comfort level with your knowledge about surgical instruments used in a C-section? _______

2. What is your current comfort level in naming in the correct order the anatomical layers encountered during a C-section? _______

3. What is your current comfort level in performing a C-section? _______

4. What is your comfort level in using appropriate suturing technique in closing each anatomical layer from a C-section? _______

5. How would you rate this C-section model and simulation? _______

6. How can we improve today’s simulation?
