# Supplementary material for: Improving Learners' Comfort With Cesarean Sections Through the Use of High-Fidelity, Low-Cost Simulation
Source: MedEdPORTAL. 2020 Feb 14;16:10878. doi: 10.15766/mep_2374-8265.10878 (PMC7062555; doi:10.15766/mep_2374-8265.10878)
Supplement: Supplementary file 1 — A. Simulation Case.docx B. CS Model Assembly and Materials.docx C. Surgical Instruments.pptx D. CS Steps and Time-out.docx E. Presimulation Survey.docx F. Postsimulation Survey.docx G. Simulation Images.docx H. Critical Actions Checklist.docx I. Debriefing Materials.docx [file mep-16-10878-s001.zip › G. Simulation Images.docx]

**Appendix G:** Simulation Event Images


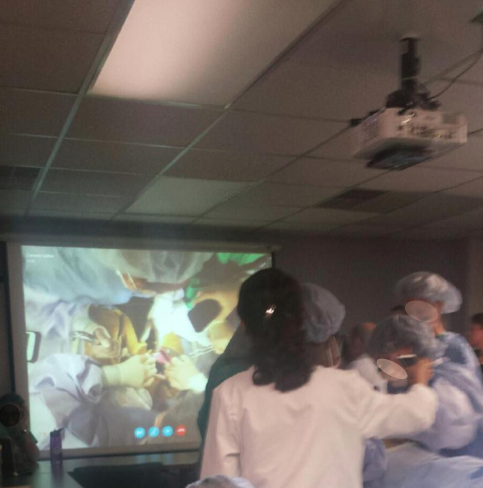


**Figure 1.** Example of media device set up to video stream the simulation. Tablet is affixed to the projector (black arrow) and directly faces the CS model.

Image is author owned


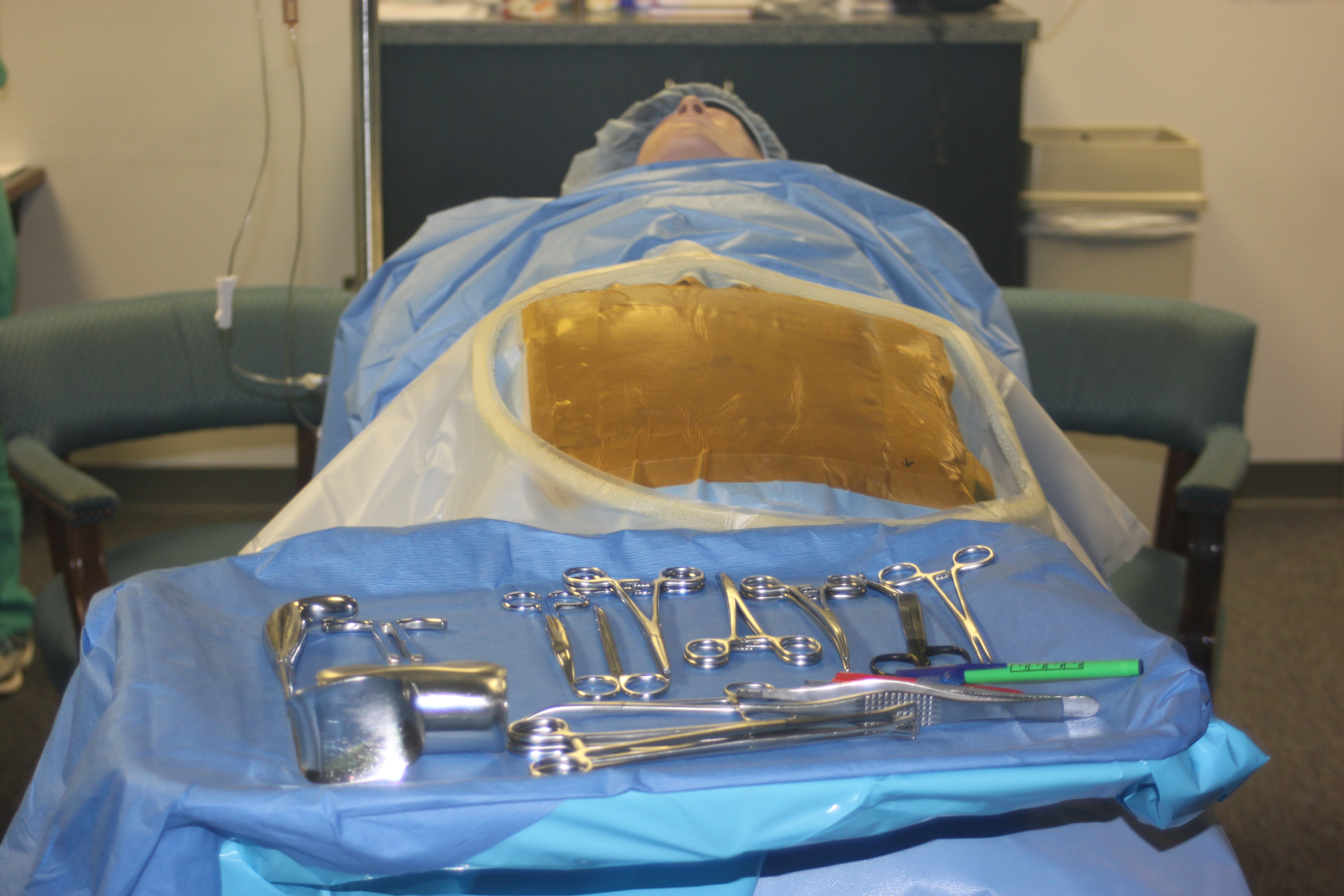


**Figure 2.** Example of setup with mannequin, Cesarean section model, drape and Mayo stand for simulation.

Image is author owned
