# Supplementary material for: Improving Learners' Comfort With Cesarean Sections Through the Use of High-Fidelity, Low-Cost Simulation
Source: MedEdPORTAL. 2020 Feb 14;16:10878. doi: 10.15766/mep_2374-8265.10878 (PMC7062555; doi:10.15766/mep_2374-8265.10878)
Supplement: Supplementary file 1 — A. Simulation Case.docx B. CS Model Assembly and Materials.docx C. Surgical Instruments.pptx D. CS Steps and Time-out.docx E. Presimulation Survey.docx F. Postsimulation Survey.docx G. Simulation Images.docx H. Critical Actions Checklist.docx I. Debriefing Materials.docx [file mep-16-10878-s001.zip › H. Critical Actions Checklist.docx]

**Appendix H:** Critical Action Checklist for Cesarean Section Simulation

Preparation

- Apply mask, shoe coverings and hair covering
- Perform a surgical scrub
- Get gowned and gloved in sterile fashion
- Prep and drape the patient in standard fashion (guided by instructor)
  - The learner will go through the motions of cleaning the abdomen per the institution’s protocol
  - The learner will drape the patient using the appropriate surgical drape

Procedure: Abdominal wall entry and delivery

- Perform a surgical time-out
- Perform an Allis test
- Call for a knife with #10 blade, verbalize “incision” to mark the start of the procedure and perform a Pfannenstiel skin incision
- Call for two medium Richardsons, a pair of Ferris-Smiths, and curved Mayos. Dissect the fascia
- Call for two Kochers and elevate the fascia off the rectus abdominis muscle
- Call for curved Mayos and dissect the remaining fascia off the rectus abdominis muscle
- Call for two Kelly clamps and Metzenbaum scissors to grasp and dissect the peritoneum. Extend the dissection bluntly
- Call for a large Richardson and a bladder blade. Retract back the abdominal wall and protect the bladder
- Call for Russian forceps and Metzenbaum scissors. Create a bladder flap (optional)
- Call for a knife with #10 blade and make a low transverse hysterotomy incision
- Call for an Allis and rupture the amniotic sac
- Remove the bladder blade and deliver the fetus using standard breech maneuvers
- Call for plastic clamps and scissors and cut the cord
- Remove the placenta via manual expression of the uterus and gentle traction on the umbilical cord
- Call for a Pennington to remove any trailing membranes from the placenta (may go through the motions for this step as no trailing membrane exists in the simulation model)
- Call for a sponge or dry lap to clear out the uterus of all debris

Procedure: Hysterotomy and abdominal wall closure

- Call for a pair of Russians and 0-Monocryl and close the hysterotomy using a running locked suture technique
- Call for two hemostats to tag the suture tails (optional) or call for suture scissors to cut the suture
- Inspect for hemostasis
- Call for two Kocher clamps and elevate the fascia to inspect for hemostasis
- Call for a medium and a small Richardson retractor, a pair of Ferris-Smiths, and 0 or 1.0 PDS. Close the fascia using a simple running suture technique. Call for suture scissors and cut the suture
- Obtain the Bovie. Inspect for hemostasis of the subcutaneous tissue and use electrosurgery where needed to establish hemostasis (may go through the motions for this step as no bleeding will be present)
- Call for Adsons and 3.0 Vicryl or Monocryl to close the subcutaneous space (when thickness is 2 cm or greater). Call for suture scissors to cut the suture
- Call for Adsons and 4.0 Vicryl to close the skin in a subcuticular fashion
