# Supplementary material for: Improving Learners' Comfort With Cesarean Sections Through the Use of High-Fidelity, Low-Cost Simulation
Source: MedEdPORTAL. 2020 Feb 14;16:10878. doi: 10.15766/mep_2374-8265.10878 (PMC7062555; doi:10.15766/mep_2374-8265.10878)
Supplement: Supplementary file 1 — A. Simulation Case.docx B. CS Model Assembly and Materials.docx C. Surgical Instruments.pptx D. CS Steps and Time-out.docx E. Presimulation Survey.docx F. Postsimulation Survey.docx G. Simulation Images.docx H. Critical Actions Checklist.docx I. Debriefing Materials.docx [file mep-16-10878-s001.zip › I. Debriefing Materials.docx]

**Appendix I:** Debriefing Materials

General Questions:

1. “What do you think you did well?”
2. “What did you find challenging?”
3. “What did you find particularly helpful?”
4. “What do you need to improve on?”

Specific Questions:

1. Why do we position the patient with a leftward tilt?

The pregnant patient is positioned with a leftward tilt during a Cesarean section (CS) to displace the gravid uterus away from the inferior vena cava. This is done to avoid compromising venous return to the heart, which could lead to maternal hypotension and fetal distress.

1. What is the purpose of the Allis test?

The Allis clamp test is performed to ensure adequate anesthesia prior to starting the CS This is performed by grabbing the patient’s skin with the Allis clamp. You want to make sure that you test the skin at the site where you will make the incision and more superiorly on the abdomen. If the patient feels pain, not just pressure, then her anesthesia is not adequate to begin the procedure.

1. Why do we use different suturing technique when closing the uterus and the fascia?

The running locking suture technique is used to close the hysterotomy because it provides better hemostasis than a simple running suture. On the other hand, a simple running suture technique is used to close the fascia as we are mostly concerned with re-approximating the tissue rather than achieving hemostasis. It is important to place the anchoring stitch behind the incision apex to prevent herniation through that space.

1. Why do we use Russian forceps to close the hysterotomy but Ferris-Smith forceps to close the facia?

Russian forceps are atraumatic instruments while Ferris-Smith forceps are traumatic instruments with teeth. The uterine tissue is highly vascular and the uterine serosa is delicate, therefore we use atraumatic forceps to handle this tissue. On the other hand, the fascia is a tougher tissue that is better handled with an instrument with teeth.

1. What is the recommendation regarding closure of the subcutaneous tissue?

Strong evidence exists to recommend closure of the subcutaneous tissue when the thickness is 2 cm or greater to decrease the risk of superficial wound disruption after a Cesarean section. ^[[1]](#endnote-1)^

1. Dahlke JD, Mendez-Figueroa H, Rouse J, Berghella V, Baxter JK, Chauhan SP. Evidence-based surgery for cesarean delivery: an updated systematic review. *Am J Obstet Gynecol*. 2013;209(4);294-306 [↑](#endnote-ref-1)
